# Rare and novel genetic variants in sporadic and familial Alzheimer’s disease: insights from the first Saudi cohort

**DOI:** 10.3389/fragi.2026.1765569

**Published:** 2026-05-08

**Authors:** Fadia El Bitar, Ghadeer Al Dawsari, Najeeb Qadi, Saad Al Rajeh, Mohamed Abouelhoda, Sahar Al Subaie, Nada Majrashi, Amna Magrashi, Hala Al Amari, Fatimah Alghamdi, Sara Abdulaziz, Bashayer Al-Mubarak, Nada Al Tassan

**Affiliations:** 1 Clinical Laboratory Department, Division of Pathology and Laboratory Medicine, Clinical Services Center of Excellence, King Faisal Specialist Hospital and Research Center, Riyadh, Saudi Arabia; 2 Precision Medicine Laboratory Department, Genomic Medicine Center, King Faisal Specialist Hospital and Research Center, Riyadh, Saudi Arabia; 3 Neuroscience Center of Excellence, King Faisal Specialist Hospital and Research Center, Riyadh, Saudi Arabia; 4 Al Habib Medical Group, Riyadh, Saudi Arabia; 5 Computational Sciences Department, Genomic Medicine Center, King Faisal Specialist Hospital and Research Center, Riyadh, Saudi Arabia; 6 Department of Clinical Laboratory Sciences, King Saud University, Riyadh, Saudi Arabia; 7 Translational Genomics Department, Genomic Medicine Center, King Faisal Specialist Hospital and Research Center, Riyadh, Saudi Arabia; 8 Bioengineering Institute, Health Sector, King Abdulaziz City for Science and Technology, Riyadh, Saudi Arabia; 9 Department of Scientific Research, Hevolution Foundation, Riyadh, Saudi Arabia

**Keywords:** Alzheimer’s disease, genes, neurodegeneration, novel variants, whole exome sequencing

## Abstract

**Background:**

Alzheimer’s disease (AD) is a complex brain disorder that is greatly affected by genetics. Next-generation sequencing (NGS) has facilitated the discovery of rare variants in new genes that may be linked to AD in different populations. However, we still know very little about the genetic makeup of AD in Saudi Arabia and other Arab populations.

**Objectives:**

This study aims to explore rare variants that are predicted to be deleterious in a group of 64 Saudi patients diagnosed with sporadic and familial Alzheimer’s disease (AD). These patients previously tested negative for mutations in genes known to cause AD and were genotyped for *APOE* alleles.

**Methods:**

We performed whole-exome sequencing (WES) on the Ion Proton platform. Then, we used our internal process for filtering, validating, and prioritizing variants.

**Results:**

Using stringent selection criteria, we identified 107 rare candidate variants with potential functional relevance. Of these, 26 (24.3%) were novel, while the remaining variants had been previously reported in public databases. Among these candidates, 33 were connected to AD, 28 to both AD and other neurodegenerative disorders (OND), 34 to OND-related functions, and 11 to broader processes like aging, inflammation, and neuronal regulation. We found rare missense variants in genes involved in important processes related to Alzheimer’s disease. These processes include mainly Aβ and Tau pathology, kinase signaling, stress response, and neuroinflammation.

**Conclusion:**

Our analysis reveals diverse genetic contributors to Alzheimer’s disease in a population that remains largely underrepresented in genomic studies. We identified candidate variants in 53% of the patients, highlighting the value of expanding AD genetics research to non-European populations.

## Introduction

Neurodegenerative disorders display substantial clinical and genetic heterogeneity. The use of next-generation sequencing (NGS), particularly whole exome sequencing (WES), has transformed our ability to detect rare, disease-associated variants in a time- and cost-efficient manner. Although exons account for about 1.5% of the human genome, they harbor approximately 85% of mutations associated with Mendelian disease. In the field of neurodegeneration, WES has significantly improved our ability to identify candidate genes linked to Alzheimer’s disease (AD), thereby enhancing our understanding of its molecular mechanisms (Tan et al., 2024; Boycott et al., 2013; Zhang et al., 2024).

Alzheimer’s disease (AD) is the most existing cause of dementia. Two primary neuropathological features characterize AD: extracellular deposition of amyloid-β peptide in the form of plaques and intraneuronal accumulation of hyperphosphorylated tau as neurofibrillary tangles (Zhang et al., 2024). Clinically, AD is stratified by age at onset. Early-onset AD (EOAD) manifests before age 65, whereas late-onset AD (LOAD) occurs thereafter and accounts for the majority of cases (Mol et al., 2022).

Although a family history occurs in 35%–62% of EOAD cases, the pattern of autosomal dominant inheritance is seen in only about 10%–15% of familial EOAD (EO-FAD). Fully penetrant single-gene mutations in *PSEN1*, *PSEN2*, and *APP* cause EO-FAD (Valdes et al., 2025). In contrast, most individuals are diagnosed with sporadic LOAD, a genetically complex disorder with heritability estimates ranging from 58% to 79%. Among the genetic susceptibility factors identified to date, the *APOE* ε4 allele remains the most robust and widely replicated risk factor for LOAD (Liu et al., 2013).

Large-scale genomic studies, including genome-wide association studies and WES, have shown that LOAD is polygenic with more than 50 genes implicated in disease susceptibility and have identified rare variants in early-onset Alzheimer’s disease (EOAD) (Zhang et al., 2024). The variants identified through WES in EOAD and LOAD have shown functional consequences on amyloid-β levels, including those in *ABCA7*, *NOTCH3*, *PLD3*, *TREM2*, and *SORL1* (Cukier et al., 2017). Other variants may protect against AD, such as *TREML2*, or promote AD pathogenesis through cleavage by active δ-secretase, as in the case of *UNC5C* (Bellenguez et al., 2017).

In the present study, we performed WES to identify rare variants and genes associated with AD. We examined a group of 64 Saudi AD patients with both sporadic and familial histories. These patients did not have mutations in known AD genes and had previously been tested for *APOE4* (El Bitar et al., 2019). Therefore, we prioritized an analysis that would increase the likelihood of identifying new candidate variants and genes implicated in Saudi AD patients.

## Materials and methods

### Participants

All patients reviewed by neurologists were recorded with complete clinical and family histories and met criteria for probable Alzheimer’s disease (AD) (Reiman et al., 2011). Our cohort consisted of 64 Saudi Alzheimer’s cases. The recruitment was conducted in accordance with the Helsinki Declaration, with written informed consent obtained after Institutional Review Board (IRB) approval at King Faisal Specialist Hospital and Research Center (RAC#2120020). Patients enrolled in this study are detailed in Table 1. They are referenced in our previous work, as these are the same cases that tested negative for mutations in the AD causative genes (*PSEN1*, *PSEN2*, and *APP*) (El Bitar et al., 2019) and for copy number variations (CNVs) in chromosomal regions harboring these genes (El Bitar et al., 2020). Patients were classified by age into EO (<65 years old) or LO (≥65 years) and by family history into familial-related AD (FMR-AD). FMR-AD includes EO-FAD, possible FAD, and AD with family history (AD-FH). Each FMR-AD category is detailed in Table 1.

**TABLE 1:** Demographic and Clinical Characteristics of 64 Saudi Alzheimer’s patients.

Category	Clinical variable	SP	EO-FAD	Poss. FAD	AD-FH	Total (n = 64)
Demographic characteristics	Number of patients	34	2	13	15	64
	Gender (F/M)	11/23	0/2	9/4	10/5	30/34
	Age at onset, mean ± SD (years)	65.9 ± 11.0	61.0 ± 4.2	59.8 ± 6.2	75.9 ± 5.7	67.3 ± 10.5
	Age at onset, range (years)	44–90	58–64	43–65	69–85	43–90
Clinical classification	Early-onset AD (<65), n (%)	17	2	4	9	32 (50.0%)
	Late-onset AD (≥65), n (%)	17	0	9	6	32 (50.0%)
Genetic characteristics	APOE ε4 carriers, n (%)	11 (32.4%)	1 (50.0%)	9 (69.2%)	9 (60.0%)	30 (46.9%)
Clinical features	Type 2 diabetes mellitus (Y/N/NR)	10/11/13	0/1/1	3/5/5	6/5/4	19/22/23
	Hypertension (Y/N/NR)	13/7/14	1/1/0	3/5/5	8/3/4	25/16/23
	Dyslipidemia (Y/N/NR)	4/17/13	0/0/2	0/5/8	4/9/2	8/31/25
	Hyperlipidemia (Y/N/NR)	3/18/13	0/0/2	1/4/8	1/12/2	5/34/25
	Cognitive impairment (Y/N/NR)	34/0/0	2/0/0	13/0/0	15/0/0	64/0/0
	Memory impairment (Y/N/NR)	34/0/0	2/0/0	13/0/0	15/0/0	64/0/0
	Agnosia (Y/N/NR)	34/0/0	2/0/0	13/0/0	15/0/0	64/0/0
	Aphasia (Y/N/NR)	8/26/0	2/0/0	8/3/2	15/0/0	33/29/2
	Ataxia (Y/N/NR)	1/33/0	0/0/2	2/4/7	1/13/1	4/50/10
Neuroimaging findings	Brain atrophy on MRI (Y/N/NR)	7/20/7	1/0/1	2/10/1	0/15/0	10/45/9
Neuropsychiatric features	Depression (Y/N/NR)	5/14/15	2/0/0	6/5/2	4/6/5	17/25/22

Keys: EO, early-onset Alzheimer’s disease (<65 years); LO, late-onset Alzheimer’s disease (≥65 years). SP, sporadic Alzheimer’s disease; FMR, familial Alzheimer’s disease–related cases. EO-FAD, early-onset familial Alzheimer’s disease (≥2 affected first-degree relatives across two generations with AAO <65 years). Poss. FAD, possible familial Alzheimer’s disease (≥1 affected first-degree relative with AAO ≥65 years or unknown). AD-FH, Alzheimer’s disease with family history (≥2 affected relatives with mixed or unknown AAO). AAO, age at onset. APOE, apolipoprotein E. Y, yes; N, no; NR, not reported.

### Whole exome sequencing capture and variants calling

#### WES read alignment and variant discovery

**FIGURE 1:**
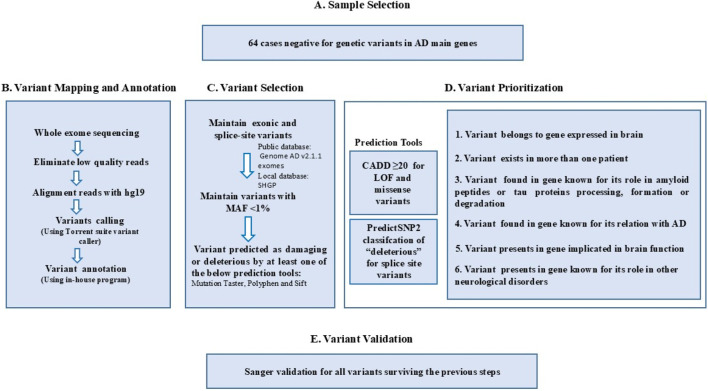
Analysis steps followed in this study: **(A)** Sample selection; **(B)** variant mapping and annotation; **(C)** Variant selection; **(D)** Variant prioritization of genes and **(E)** Variant validation. Genetic variants include single nucleotide variant (SNV) and copy number variation (CNV).

We performed whole-exome capture for our study cases using the AmpliSeq whole-exome kit. Briefly, peripheral blood DNA was extracted following the manufacturer’s instructions with the PureLink Genomic DNA kit (Thermo Fisher, Carlsbad, CA, United States). Genomic DNA (100 ng per sample) was sequenced on the Ion Proton TM System platform. Next, high-quality reads with adequate coverage (≥10×) underwent quality control (QC) to remove low-quality sequences, followed by mapping and alignment to the UCSC human reference genome (hg19) (http://genome.ucsc.edu/).We performed variant calling using the Torrent Suite Variant Caller (Life Technologies, Carlsbad, CA, United States). The Variant calling plugin was set to include variants with a minimum coverage of 20X and a minimum variant calling quality score of 50. Variants were then annotated with ANNOVAR (http://annovar.openbioinformatics.org). Figure 1 summarizes the WES workflow used in our study. Variant is filtered that minimum depth 20 for SNPs and 30 for Indels to account for calling errors.

#### Variant selection

To maximize the likelihood of identifying candidate variants associated with Alzheimer’s disease, we considered variants across all chromosomes. Variant filtration included both homozygous and heterozygous variants in familial and sporadic cases (Supplementary Table S2). Filtration and validation of variants were performed as described earlier (Al-Mubarak et al., 2017). First, functional variants (LoF and missense) were selected, followed by the selection of rare variants with a minor allele frequency (MAF) below 1% from the local Saudi Human Genome Program (SHGP) database and the international database (gnomAD (Karczewski et al., 2020)). At the time of our analysis, the SHGP database consisted of NGS data from 13,000 exomes of ethnically matched local controls. Then, multiple variant effect prediction tools were used to evaluate the potentially damaging impact of variants. Widely used *in silico* tools, such as Mutation Taster (Schwarz et al., 2014), PolyPhen (Adzhubei et al., 2013), SIFT (Sim et al., 2012) were employed for the relevant functional categories (Figure 1C). Novel variants were defined as variants absent from major public variant databases, including gnomAD, ClinVar and dbSNP, at the time of analysis, indicating that they had not been previously reported in population or disease-associated variant repositories (Supplementary Table S3).

#### Variant prioritization and validation

We further narrowed our selection to variants that fulfilled the standard of prediction tools CADD v1.6 (Rentzsch et al., 2021) of score ≥ to 20 and PredictSNP2 classification of “deleterious” for splice site variants (Bendl et al., 2016). We prioritized the resulting variants based on displaying at least one of six criteria as follows: 1. Presence in a gene expressed in the brain, confirmed by public repositories (GTEx, BioGPS, Illumina Body Map, and Serial Analysis of Gene Expression), which is mandatory for all variants in addition to one of the other listed criteria, 2. Presence in more than one patient, 3. Presence in a gene known for its role in amyloid peptides or tau proteins processing, formation, or degradation, 4. Presence in a gene associated with AD, 5. Presence in a gene implicated in brain function (such as ageing, memory, synaptic plasticity) and 6. Presence in a gene known for its implication in other neurological diseases (Figure 1D). The resulting short-listed variants were validated for their actual presence (nomenclature and genotype) by Sanger sequencing (Figure 1E).

#### Variant interpretation and ACMG/AMP classification

Prioritized variants were systematically evaluated according to the American College of Medical Genetics and Genomics and the Association for Molecular Pathology (ACMG/AMP) guidelines (Richards et al., 2015). For each variant, applicable ACMG/AMP evidence criteria (e.g., PVS1, PM2, PP3, BP4) were carefully assessed and assigned where relevant. Based on the combined evidence, variants were categorized as Pathogenic, Likely Pathogenic, Variants of Uncertain Significance (VUS), Likely Benign, or Benign. Given the complex and multifactorial nature of Alzheimer’s disease, ACMG classification was applied as a standardized interpretative framework to support variant evaluation rather than as definitive evidence of clinical pathogenicity.

## Results

### Selected variants

WES generated a total of 2,871,742 variants that passed our quality control filters, including 2,744,560 single-nucleotide variants (SNVs) and 127,182 insertions and deletions (indels) (Supplementary Table S1). Each exome contains an average of 44,871 total variants (Supplementary Table S1). In the filtration step, we considered both homozygous and heterozygous variants (Figure 1), as our cohort included 32 sporadic cases and 32 familial-related cases (Table 1).

**FIGURE 2:**
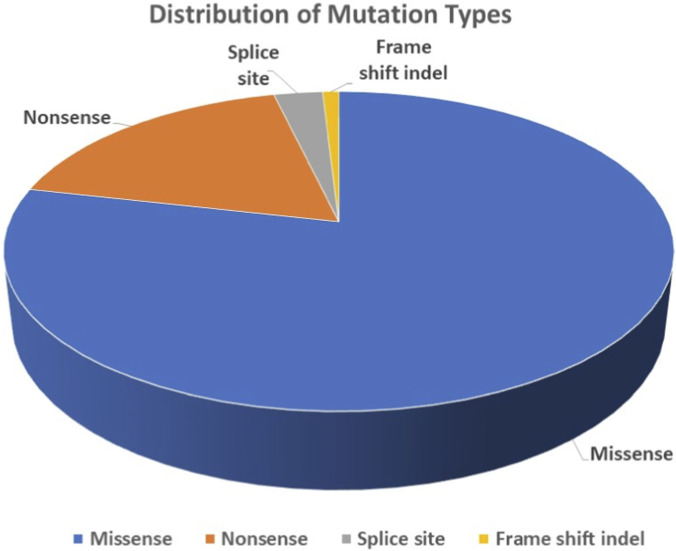
Overview of 107 selected genetic variations found in the present study.

**FIGURE 3:**
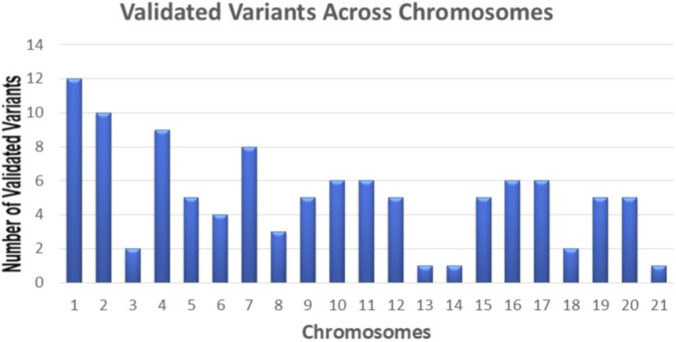
Distribution of validated variants across chromosomes. Several validated variants identified in our study were mapped to genes distributed across the genome

We selected variants located in exonic or splice-site regions with MAF <1% in population databases (SHGP and gnomAD v3). The next step involved selecting variants designated as deleterious by at least one of the prediction tools (Figure 1C). We further narrowed our selection to variants that met the standard of prediction tools (CADD score ≥20 and PredictSNP2 classification of “deleterious” for splice site variants) (Figure 1D), in addition to having at least one of the criteria for variant prioritization (Figure 1D). Using the described pipeline following stringent prioritization, we identified 107 deleterious variants (Supplementary Table S2), which were confirmed as true positives by Sanger sequencing (Figure 1D). The majority of these variants are classified as missense (84/107), followed by nonsense (19/107), splice sites (3/107), and frameshift (1/107) (Supplementary Table S2, Figure 2). The total variants were distributed across 106 different genes on various chromosomes (Figure 3). The identified variants have CADD scores ranging from 20 to 58, with 39 exhibiting CADD scores ≥30 (Supplementary Table S2). Of the 107 rare candidate variants, 26 (24.3%) were novel, while 81 (75.7%) had been previously reported in public databases. ClinVar annotation showed that 77 variants (72.0%) were not previously reported, whereas 28 (26.2%) were classified as variants of uncertain significance (VUS), and only one variant each (0.9%) was annotated as Pathogenic and Likely Pathogenic. According to ACMG/AMP classification applied in this study, the majority of variants were categorized as VUS, with a smaller proportion classified as Likely Pathogenic or Pathogenic, and one variant as Likely Benign. Notably, none of the variants classified as Pathogenic or Likely Pathogenic had been previously reported in AD or OND based on a targeted PubMed search (Supplementary Table S3).

### Candidate variants

The variants from restricted selection appeared in 34 of the 64 patients. They were spread across 16 early-onset Alzheimer’s disease (EO) cases and 18 late-onset Alzheimer’s disease (LOAD) cases. Of these, 20 were familial related (FMR) and 14 were sporadic (SP) (Supplementary Table S2). The *APOE* ε3/ε4 risk allele was present in 17 patients. Additionally, two cases, AD 26 and AD 45, were carriers of *APOE* ε4/ε4 (Supplementary Table S2). We observed five or more variants in 8 of the 34 patients (Supplementary Table S2).

We identified a group of rare missense variants in several genes known to participate in key molecular pathways associated with Alzheimer’s disease (AD) and other neurodegenerative disorders (OND) (Table 2). These genes displayed roles in multiple AD-related biological processes, including amyloid-β metabolism, tau phosphorylation, kinase signaling, calcium homeostasis, neuroinflammation, and synaptic regulation. Within the amyloid-β pathway, we pointed out *CD36*, *DAB1*, *GRK5*, and *CALHM1* for their roles in promoting Aβ clearance. We detected variants also in *LIMK1*, *RELN*, *PRKCG*, *TCF7L1*, and *N4BP3*, which are related to tau pathology and cytoskeletal organization.

**TABLE 2:** Representative genes directly related to AD/OND, grouped by functional pathways.

Functional Subgroup	Gene Symbol	Category	Key Role	Reference
Aβ (Amyloid-β) Pathway	*CD36*	AD	Microglial uptake of Aβ, lipid metabolism	El Khoury et al. (2003), Dobri et al. (2021)
	*DAB1*	AD	Reelin–APP signaling, modulates amyloid deposition	Homayouni et al. (1999)
	*GRK5*	AD	Kinase linked to Aβ accumulation and synaptic dysfunction	Cheng et al. (2010)
	*CALHM1*	AD	Ca^2+^ homeostasis, affects Aβ levels	Dreses-Werringloer et al. (2008)
Tau Pathology/Cytoskeletal Regulation	*LIMK1*	AD	Actin/tau pathway; tau phosphorylation and spine dynamics	Heredia et al. (2006)
	*RELN*	AD	Reelin influences tau phosphorylation and amyloid signaling	Ohkubo et al. (2003), Hoe et al. (2009)
	*PRKCG*	AD-OND	PKCγ; synaptic plasticity and APP/tau regulation	Lordén and Newton (2021), Skovronsky et al. (2000), Boyce and Shea (1997)
	*TCF7L1*	AD	Wnt pathway upstream of tau kinases	Riise et al. (2015)
	*N4BP3*	AD-OND	Regulates axonal and dendritic growth via Nedd4 interaction; essential for neurite outgrowth and synaptic connectivity	Schmeisser et al. (2013)
Kinase Signaling/Stress Response	*PDK1*	AD	Kinase hub intersecting with tau kinase signaling and metabolism	Yang et al. (2022)
	*FKBP5*	AD-OND	Chaperone regulating tau folding, stress pathways	Blair et al. (2013)
Calcium/Channel Dysregulation	*CACNA2D4*	AD	Ca^2+^ channel subunit, APP/tau dysregulation	Daschil et al. (2013)
	*KCNMA1*	AD	BK (KCa1.1) channel suppressed by Aβ/APP interaction, leading to neuronal hyperexcitability and synaptic dysfunction in AD models	Yamamoto et al. (2021)
Neuroinflammation/Microenvironment	*NFKBIE*	AD	Inflammatory regulator; tau phosphorylation	Wang T. et al. (2022)
​	*MMP2*	AD-OND	ECM remodeling, BBB integrity, plaque environment	Wang et al. (2020)
Synaptic/Dendritic Spine Regulation	*KALRN*	AD	Regulates dendritic spine dynamics, affected by Aβ oligomers	Xie et al. (2019)
	*JADE2*	AD	Histone acetylation regulator controlling RAC1 transcription; essential for hippocampal synaptic plasticity and memory	Fan et al. (2022)
Inflammation/Metabolic Regulation	*PAPPA*	AD	Metalloproteinase regulating IGF signaling; deletion reduces amyloid burden and improves cognition in AD mouse models	Bale et al. (2024)

In addition, variants were detected in genes implicated in kinase signaling and stress response, including *PDK1* and *FKBP5,* which intersect with metabolic and tau-related kinase networks. We observed variants in genes contributing to calcium and ion channel regulation. Among them are *CACNA2D4* and *KCNMA1*, which are involved in altered neuronal excitability and calcium balance. We also observed variants in genes related to neuroinflammation and extracellular matrix remodeling *(NFKBIE* and *MMP2*). Finally, we identified variants in genes linked to synaptic and dendritic spine regulation (*KALRN*, *JADE2*), lipid and metabolic regulation (*PAPPA*) (Table 2; Supplementary Table S2).

Among the rare variants with no known link to Alzheimer’s disease (AD) or other neurodegenerative disorders (OND), we found variants in genes that may participate in AD-related biological processes. These genes include those involved in aging (*DHX57*) and in memory and cognitive control (*ACSS2*) (Supplementary Table S2).

## Discussion

Advances in next-generation sequencing have expanded our ability to detect rare, potentially deleterious variants underlying complex neurodegeneration such as Alzheimer’s disease (AD) (Tan et al., 2024; Boycott et al., 2013). In this first whole-exome analysis of familial and sporadic Saudi AD cases, we applied a multistep filtering strategy encompassing all inheritance modes (Figure 1). We identified mainly missense variants, with additional loss-of-function (LoF) classes (nonsense, frameshift, splice) enriched in biologically relevant pathways. Of 107 rare variants, 72 are part of genes with known or putative roles in convergent signals in Aβ handling, tau/cytoskeletal regulation, ion/calcium homeostasis, synaptic plasticity, kinase/stress pathways, and neuroinflammation/metabolic milieu (Table 2; Supplementary Table S2).

### Convergence on Aβ processing and clearance

We identified variants in *CD36*, *DAB1*, *GRK5*, and *CALHM1* (Table 2; Supplementary Table S2: AD-14, AD-59, AD-70, AD-11). These genes contribute in disrupting Aβ clearance and altering APP processing. For instance, *CD36* is implicated in microglial uptake of Aβ and lipid handling (El Khoury et al., 2003; Dobri et al., 2021), whereas *DAB1* links Reelin–APP signaling pathways and influences amyloid deposition (Homayouni et al., 1999). *GRK5* has also been associated with Aβ buildup and synaptic dysfunction (Cheng et al., 2010), and *CALHM1* helps regulate calcium flux, which, in turn, can shape Aβ levels (Dreses-Werringloer et al., 2008). Interestingly, several of these variants are found in individuals carrying *APOE* ε3/ε4 or ε4/ε4 genotypes, for example, *CALHM1* E233X in AD-11, *GRK5* R394Q in AD-59, *DAB1* C130Y in AD-70, and *CD36* A419D in AD-14. Overall, these findings suggest *APOE*–ε4–related risk, along with regulators in Aβ-related genes, indicating a compounding impact on disease pathology (Bracher-Smith et al., 2022; Huq et al., 2021).

### Tau, actin cytoskeleton, and spine stability

Multiple variants in *LIMK1*, *RELN*, *PRKCG*, *TCF7L1*, and *N4BP3* (Table 1; Supplementary Table S2: AD-14 *LIMK1* V512M; AD-18 *RELN* G181R; AD-17 *PRKCG* A461T; AD-45 *TCF7L1* C368G; AD-32 *N4BP3* L297V) give an indication of the distinct influences of these genes on actin dynamics and the interaction between tau and kinases. Specifically, *LIMK1* may alter regulation of actin and tau pathways as well as affects dendritic spine morphology (Heredia et al., 2006); *RELN* may impact RELN signaling, tau phosphorylation, and amyloid pathways (Ohkubo et al., 2003; Hoe et al., 2009); *PRKCG* could influence APP and tau regulation as well as synaptic plasticity (Lordén and Newton, 2021; Skovronsky et al., 2000; Boyce and Shea, 1997); *TCF7L1* may place the Wnt signaling pathway upstream of tau kinases (Riise et al., 2015). Additionally, *N4BP3* may affect neurite outgrowth and synaptic connectivity (Schmeisser et al., 2013). Collectively, these findings suggest that structural synaptopathy could contribute to cognitive decline (Meftah and Gan, 2023).

### Ion channels and calcium signaling

We identified missense variants in *CACNA2D4* and *KCNMA1* (Table 2; Supplementary Table S2: AD-49 *CACNA2D4* R355Q; AD-16 *KCNMA1* S59F). Changes in these genes suggest Ca^2+^ dysregulation and neuronal hyperexcitability, which can influence *APP* processing and tau kinase activity. *CACNA2D4* shows an impact on Ca^2+^ channel activity, APP, and tau dysregulation (Daschil et al., 2013); *KCNMA1* (BK channel) is suppressed by Aβ and APP interaction in AD models, promoting synaptic dysfunction (Yamamoto et al., 2021). Additional transporter/channel-adjacent signals include splice in *SLC30A7* (AD-59) (Niu et al., 2022; Yang et al., 2016) and truncation in *SLC7A2* (AD-40) (Li Y. et al., 2020).

### Kinase hubs and stress-response signaling

Our analysis revealed missense variants in *PDK1* and *FKBP5* (Table 2; Supplementary Table S2: AD-48 *PDK1* G354C; AD-8 *FKBP5* P246L). These genes lead to influence the PI3K–AKT pathway and the glucocorticoid/chaperone systems. Both of which intersect with *APP* processing, tau phosphorylation, and neuronal survival. *PDK1* acts as central kinase that correlates metabolic regulation with tau-related signaling (Yang et al., 2022). *FKBP5* interferes in tau folding and responds to cellular stress, and it has also been connected to both AD and depression-related traits in tau folding and stress pathways (Blair et al., 2013). Additional stress/kinase genes in Supplementary Table S2, such as *DGKD* in AD-45, reinforce a broad signal-transduction burden (Lin et al., 2006).

### Synaptic scaffolding, autophagy, and chromatin control

Variants in *KALRN* and *JADE2* (Table 2; Supplementary Table S2: AD-60 *KALRN* E454Q; AD-70 *JADE2* splice) are parts of genes that point to defects in dendritic spine remodeling and histone-dependent transcriptional control of synaptic programs (*JADE2* → RAC1). *KALRN* is affected by Aβ oligomers and regulates spine dynamics (Xie et al., 2019); *JADE2* controls histone acetylation and hippocampal synaptic plasticity/memory (Fan et al., 2022). Additional synaptic/autophagy regulators include *WDFY3* and *SHC3* (AD-78; *WDFY3* C3269Y; *SHC3* V521I) and *PUM2* (AD-23), supporting altered cargo trafficking and autophagic flux in proteinopathy (Bailus et al., 2021; Zhu et al., 2020).

### Neuroinflammation and tissue microenvironment

Signals from *NFKBIE* and *MMP2* (Table 2; Supplementary Table S2: AD-43 *NFKBIE* A57V; AD-10 *MMP2* S102G) suggest that they play roles in NF-κB-mediated inflammatory control and in remodeling of the extracellular matrix (ECM) and blood-brain barrier (BBB), respectively. *NFKBIE* is an inflammatory regulator that modulates NF-κB signaling and can affect tau phosphorylation (Wang C. et al., 2022). On the other hand, *MMP2* influences the ECM, impacting BBB integrity and the structure of the plaque microenvironment (Wang et al., 2020). The *SERPINB1* variant (AD-18 S378Y) further links innate immune responses—through protease inhibition—to amyloid metrics (Calligaris et al., 2015; Hernandez-Guillamon et al., 2015). *PAPPA* (AD-18 D1461N) connects IGF signaling with amyloid burden and cognition (Bale et al., 2024).

### Metabolic and circadian modifiers

Several variants are present in genes involved in energy metabolism and circadian regulation, including *GOT1* (AD-18 A84T; AD/OND links) (Li W. X. et al., 2020; Ray et al., 2008), *CRYM* (AD-59 frameshift) (Aksoy et al., 2022), *ACSS2* (AD-55 R110Q) (Mews et al., 2017), and *CLOCK* (AD-83 R66G) (Yang et al., 2016; Lamont et al., 2007; Shkodina et al., 2022) (Supplementary Table S2). The implication of these genes seems in neurotransmitter cycling, thyroid-dependent metabolic regulation, acetyl-CoA generation, and histone acetylation related to memory and circadian rhythm entrainment. Together, these insights reflect a mechanistic rationale for metabolic and chronobiological influences on cognitive decline in this cohort (Musiek and Holtzman, 2016; Peixoto and Abel, 2013).

### Presence of multiple rare candidate variants in individual patients

The identification of multiple rare variants within the same patient may suggest a cumulative genetic effect on disease susceptibility. Importantly, the coexistence of biologically relevant variants such as the *PRKCG* variant identified in patient AD-17 (Lordén and Newton, 2021; Skovronsky et al., 2000; Boyce and Shea, 1997) and the *RELN* variant in patient AD-18 (Yu et al., 2016), the *ABCA8* variant in patient AD-50 (Kim et al., 2008), together with additional rare variants of currently unknown function within the same individuals (Supplementary Table S2), highlights the potential for cumulative or modifying effects. These genes displayed roles in synaptic signaling, neuronal function, and lipid homeostasis, processes highly relevant to neurodegeneration. Although the pathogenic contribution of the accompanying rare variants remains to be determined, their presence within the same genetic background suggests they may act collectively or as genetic modifiers. Future functional studies will be essential to explore their biological roles and precisely whether their combined effects contribute to the genetic complexity and heterogeneity of AD, further supporting a potential oligogenic model of disease susceptibility.

### Cross-disorder genetic overlap and phenotype modifiers

Consistent with pleiotropy across brain disorders, Supplementary Table S2 documents variants in genes associated with PD, LBD, FTD, ALS, MDD, ASD, and SCZ (e.g., *PLCG2*, *SYBU*, *GPR158*, *GOT1*, *DOCK10*, *PRKCG*, *FLNC*, *CLOCK*, *ABLIM1*, *SACS*), with representative evidence in the table for AD as well as other neurodegenerative diseases (van der Lee et al., 2019; Bisht et al., 2020; Lobanov et al., 2022; Jiang et al., 2019). These variants affect genes implicated in synaptic and axonal structure, immune signaling, mitochondrial–metabolic pathways, and circadian regulation, mirroring cross-disorder molecular modules described in large-scale multi-omic analyses of neuropsychiatric and neurodegenerative conditions (Gandal et al., 2018; Ramanan and Saykin, 2013; Wang et al., 2018). Together with evidence that circadian disruption influences neurodegenerative phenotypes (Musiek and Holtzman, 2016). These findings reinforce the view that such shared biological pathways modulate clinical expression, including depression, Parkinsonism, and cognitive fluctuation within AD.

## Conclusion

The insights from our findings are limited by the relatively small cohort size and the absence of formal statistical enrichment or rare-variant burden testing. However, all identified variants were confirmed to be rare by comparison with more than 13,000 Saudi SHGP controls. Additional limitations include incomplete segregation analysis and the lack of functional validation for individual variants.

Future work is recommended to target segregation analysis in available relatives, rare-variant burden testing against Saudi controls with *APOE*-stratified analyses, and functional characterization of key candidate variants, including *CALHM1* E233X, *GRK5* R394Q, *JADE2* splice-site, *SLC30A7* splice-site, and *WDFY3*/*SHC3* missense variants. Multi-omic integration, when available, with patient-derived molecular data and more detailed clinical phenotyping, will further help contextualize the biological relevance of these findings.

## Data Availability

The variant data for this study have been deposited in the European Variation Archive (EVA) at EMBL-EBI under accession number PRJEB112178 and is publicly available at https://www.ebi.ac.uk/eva/?eva-study=PRJEB112178.
