# Supplementary material for: Rare and novel genetic variants in sporadic and familial Alzheimer’s disease: insights from the first Saudi cohort
Source: Front Aging. 2026 May 8;7:1765569. doi: 10.3389/fragi.2026.1765569 (PMC13194459; doi:10.3389/fragi.2026.1765569)
Supplement: Supplementary file 1 [file Supplementaryfile1.docx]

**Rare and Novel Genetic Variants in Sporadic and Familial Alzheimer’s Disease: Insights from the First Saudi Cohort**

**Supplementary Materials**

**S1 Table. All the detected variants identified in our study before applying the filtering pipeline.**

**S2 Table. Total variants selected in the present study after filtration and prioritization.**

**S3 Table: Rare candidate variants identified in the AD cohort with ClinVar annotation and ACMG/AMP classification.**

**S1 Table. All the detected variants identified in our study before applying the filtering pipeline.**

| **No.** | **Proband ID (Gender)** | **Total Variant Count** | **Total SNV Count** | **Total Indel Count** |
| --- | --- | --- | --- | --- |
| **1** | **AD-8(F)** | **58,803** | **55,758** | **3045** |
| **2** | **AD-9(F)** | **39064** | **37569** | **1495** |
| **3** | **AD-10(M)** | **39422** | **37937** | **1485** |
| **4** | **AD-11(F)** | **39179** | **37659** | **1520** |
| **5** | **AD-12(M)** | **37587** | **36138** | **1449** |
| **6** | **AD-13(F)** | **39410** | **37884** | **1526** |
| **7** | **AD-14(F)** | **38768** | **37135** | **1633** |
| **8** | **AD-15(F)** | **38565** | **37017** | **1548** |
| **9** | **AD-16(F)** | **41458** | **39856** | **1602** |
| **10** | **AD-17(M)** | **38905** | **37441** | **1464** |
| **11** | **AD-18(F)** | **39497** | **37987** | **1510** |
| **12** | **AD-19(F)** | **40131** | **38577** | **1554** |
| **13** | **AD-20(M)** | **40540** | **38950** | **1590** |
| **14** | **AD-22(M)** | **38877** | **37421** | **1456** |
| **15** | **AD-23(F)** | **38928** | **37392** | **1536** |
| **16** | **AD-24(M)** | **39267** | **37772** | **1495** |
| **17** | **AD-25(M)** | **54793** | **51926** | **2867** |
| **18** | **AD-26(M)** | **54537** | **51644** | **2893** |
| **19** | **AD-27(M)** | **56825** | **53661** | **3164** |
| **20** | **AD-28(M)** | **57457** | **54512** | **2945** |
| **21** | **AD-30(M)** | **38361** | **36854** | **1507** |
| **22** | **AD-31(F)** | **39042** | **37227** | **1815** |
| **23** | **AD-32(F)** | **39335** | **37782** | **1553** |
| **24** | **AD-33(F)** | **40868** | **39262** | **1606** |
| **25** | **AD-34(F)** | **39644** | **38146** | **1498** |
| **26** | **AD-35(F)** | **39580** | **38049** | **1531** |
| **27** | **AD-37(M)** | **39648** | **38090** | **1558** |
| **28** | **AD-38(M)** | **38810** | **37300** | **1510** |
| **29** | **AD-39(F)** | **39119** | **37589** | **1530** |
| **30** | **AD-40(F)** | **41071** | **39489** | **1582** |
| **31** | **AD-41(M)** | **38796** | **37272** | **1524** |
| **32** | **AD-42(F)** | **58455** | **55260** | **3195** |
| **33** | **AD-43(F)** | **58153** | **55089** | **3064** |
| **34** | **AD-44(M)** | **39242** | **37708** | **1534** |
| **35** | **AD-45(M)** | **40908** | **39333** | **1575** |
| **36** | **AD-46(F)** | **39492** | **37949** | **1543** |
| **37** | **AD-48(F)** | **38638** | **37193** | **1445** |
| **38** | **AD-49(F)** | **39142** | **37638** | **1504** |
| **39** | **AD-50(F)** | **39285** | **37799** | **1486** |
| **40** | **AD-51(M)** | **39130** | **37634** | **1496** |
| **41** | **AD-52(F)** | **39177** | **37716** | **1461** |
| **42** | **AD-53(F)** | **41665** | **40173** | **1492** |
| **43** | **AD-54(F)** | **40299** | **38738** | **1561** |
| **44** | **AD-55(F)** | **40475** | **39047** | **1428** |
| **45** | **AD-56(M)** | **39910** | **38412** | **1498** |
| **46** | **AD-57(M)** | **56350** | **53138** | **3212** |
| **47** | **AD-58(F)** | **56266** | **53317** | **2949** |
| **48** | **AD-59(M)** | **56293** | **53339** | **2954** |
| **49** | **AD-60(M)** | **63505** | **60232** | **3273** |
| **50** | **AD-70(F)** | **38127** | **36759** | **1368** |
| **51** | **AD-71(M)** | **38437** | **36993** | **1444** |
| **52** | **AD-72(M)** | **38750** | **37277** | **1473** |
| **53** | **AD-73(M)** | **39253** | **37693** | **1560** |
| **54** | **AD-74(M)** | **55596** | **52530** | **3066** |
| **55** | **AD-75(M)** | **54032** | **51189** | **2843** |
| **56** | **AD-76(F)** | **54521** | **51812** | **2709** |
| **57** | **AD-77(M)** | **55903** | **52961** | **2942** |
| **58** | **AD-78(M)** | **54991** | **52138** | **2853** |
| **59** | **AD-79(F)** | **59446** | **56152** | **3294** |
| **60** | **AD-80(F)** | **57765** | **54689** | **3076** |
| **61** | **AD-81(F)** | **55931** | **53031** | **2900** |
| **62** | **AD-82(M)** | **55606** | **52701** | **2905** |
| **63** | **AD-83(M)** | **37679** | **36221** | **1458** |
| **64** | **AD-84(F)** | **43033** | **41403** | **1630** |
|  | **Total variant** | **2,871,742** | **2,744,560** | **127,182** |
|  | **Mean** | **44870.96875** | **42883.75** | **1987.21875** |
|  |  |  |  |  |
| Keys: F, Female; M, Male; SNV, Small Nucleotide Variants. | | | |  |

**S2 Table: Total variants selected in the present study after filtration and prioritization.**

| **Proband ID/**  **Gender** | **AAO** | **FMR/SP** | **APOE**  **Genotype** | **Gene** | **Chr#:**  **Start-End** | **Base Change** | **AA Change** | **Variant**  **type** | **CADD score** | **Zygosity** | **AD/ Ref** | **OND/**  **Ref** | **Other**  **Functions** | **#P** |
| --- | --- | --- | --- | --- | --- | --- | --- | --- | --- | --- | --- | --- | --- | --- |
| **AD-8(F)** | **59** | **FMR** | **Ɛ3/ Ɛ4** | ***DOCK8***  ***NM__***  ***001190458*** | **Chr9:**  **396806-**  **396806** | **c.2692 G>A** | **p. V898I** | **missense** | **21.8** | **Het** | **_** | **MS, GL/[1]** | **_** | **1,2,6** |
|  |  |  |  | ***FKBP5 NM_***  ***001145777*** | **Chr6:**  **35554823-35554823** | **c.737**  **C>T** | **p. P246L** | **missense** | **20.5** | **Het** | **AD/**  **[2]** | **HD/**  **[2],**  **MDD/**  **[3]** | **_** | **1,4,6** |
|  |  |  |  | ***MAN1C1***  ***NM__***  ***020379*** | **Chr1:**  **26090364-26090364** | **c.1048-1 G>A** | **-** | **splice site** | **27.9** | **Het** | **_** | **PD/**  **[4]** | **_** | **1,6** |
| **AD-10(M)** | **63** | **SP** | **Ɛ3/ Ɛ3** | ***MMP2***  ***NM_***  ***001127891*** | **Chr16:**  **55518001-55518001** | **c.304 A>G** | **p. S102G** | **missense** | **24.8** | **Het** | ***AD/[5]** | **RDD [6]** | **_** | **1,3,4,6** |
| **AD-11(F)** | **64** | **FMR** | **Ɛ3/ Ɛ4** | ***CALHM1 NM_***  ***001001412*** | **Chr10:**  **105215363-105215363** | **c.697 G>T** | **p. E233X** | **nonsense** | **37** | **Het** | **_** | **EP/**  **[7]** | **_** | **1,6** |
|  |  |  |  | ***GLP2R NM_004246*** | **Chr17:**  **9729394-9729394** | **c.14**  **C>A** | **p. S5X** | **nonsense** | **22.3** | **Het** | **AD/**  **[8]** | **CD, PD/[8]** | **_** | **1,4,6** |
|  |  |  |  | ***PDE4DIP* NM_001002811** | **Chr1:**  **144921867-144921867** | **c.1651 C>T** | **p. Q551X** | **nonsense** | **39** | **Het** | **AD/**  **[9]** | **FTD, VaD/**  **[10]** | **_** | **1,2,4,6** |
| **AD-13(F)** | **64** | **FMR** | **Ɛ3/ Ɛ4** | ***NBAS NM_015909*** | **Chr2:**  **15470842-15470842** | **c.4227 G>A** | **p. W1409X** | **nonsense** | **43** | **Het** | **AD/**  **[11]** | **_** | **_** | **1,4** |
| **AD-14(F)** | **63** | **FMR** | **Ɛ3/ Ɛ4** | ***CD36 NM_001289908*** | **Chr7:**  **80303417-80303417** | **c.1256 C>A** | **p. A419D** | **missense** | **20.1** | **Hom** | ***AD/**  **[12]** | **_** | **_** | **1,3** |
|  |  |  |  | ***DHX57 NM_198963*** | **Chr2:**  **39025584-39025584** | **c.4018 G>A** | **p. V1340M** | **missense** | **25.8** | **Het** | **_** | **_** | **Aging/**  **[13]** | **1,4** |
|  |  |  |  | ***LIMK1 NM_001204426*** | **chr7:**  **73535234-73535234** | **c.1534 G>A** | **p. V512M** | **missense** | **25** | **Het** | ***AD/**  **[14]** | **_** | **_** | **1,3** |
| **AD-16(F)** | **59** | **FMR** | **Ɛ3/ Ɛ4** | ***KCNMA1 NM_*** ***NM_001014797*** | **Chr10:**  **79397225-79397225** | **c.176 C>T** | **p. S59F** | **missense** | **24** | **Het** | **_** | **PNK,**  **EP/**  **[15]** |  | **1,6** |
|  |  |  |  | ***PLCG2 NM_002661*** | **Chr16:**  **81939026-81939026** | **c.1381 C>T** | **p. R461X** | **nonsense** | **38** | **Het** | **AD/**  **[16]** | **FTD, LBD**  **/[17]** | **_** | **1,4,6** |
|  |  |  |  | ***PTDSS1 NM_014754*** | **Chr8:**  **97318687-97318687** | **c.910 A>T** | **p. T304S** | **missense** | **26.8** | **Het** | **_** | **DD/ [18]** | **_** | **1,6** |
| **AD-17(M)** | **58** | **FMR** | **Ɛ3/ Ɛ3** | ***SLC17A6 NM_020346*** | **chr11:**  **22397632-22397632** | **c.1279 A>G** | **p. I427V** | **missense** | **30** | **Het** | **_** | **PD/**  **[19]** | **_** | **1,6** |
|  |  |  |  | ***STEAP4 NM_024636*** | **Chr7:**  **87913436-87913436** | **c 149 G>A** | **p. R50Q** | **missense** | **22.3** | **Het** | **_** | **_** | **Inflammatory process/[20]** | **1,5** |
|  |  |  |  | ***ADAMTS8 NM_007037*** | **Chr11:**  **130286172-130286172** | **c.1150 G>A** | **p. G384R** | **missense** | **32** | **Het** | **_** | **_** | **Inflammatory process/[21]** | **1,5** |
|  |  |  |  | ***TMEM87A NM_001286487*** | **Chr15:**  **42519008-42519008** | **c.1216 C>G** | **p. Q406E** | **missense** | **33** | **Het** | **_** | **_** | **Memory and cognitive function/[22]** | **1,5** |
|  |  |  |  | ***RASL12 NM_016563*** | **Chr15: 65350898-**  **65350898** | **c.292 T>C** | **p. Y98H** | **missense** | **24.2** | **Hom** | **AD/**  **[23]** | **_** | **_** | **1,4** |
|  |  |  |  | ***MACF1 NM_012090*** | **Chr1:**  **39719970-39719970** | **c.373 C>G** | **p. P125A** | **missense** | **29** | **Het** | **AD/**  **[24]** | **FP/**  **[25]** | **_** | **1,4,6** |
|  |  |  |  | ***ASTN1 NM_004319*** | **Chr1:**  **176833497-176833497** | **c.3808 C>T** | **p. R1270W** | **missense** | **31** | **Het** | **_** | **ID/**  **[26]** | **_** | **1,6** |
|  |  |  |  | ***FH NM_000143*** | **Chr1:**  **241667401-241667401** | **c.1049 G>A** | **p. R350Q** | **missense** | **34** | **Het** | **AD/**  **[27]** | **_** | **_** | **1,4,** |
|  |  |  |  | ***TCERG1 NM_006706*** | **Chr5:**  **145858109-145858109** | **c.1655 G>A** | **p. R552H** | **missense** | **36** | **Het** | **_** | **HD/**  **[28]** | **_** | **1,6** |
|  |  |  |  | ***LONP1 NM_004793*** | **Chr19:**  **5719824-5719824** | **c.320 C>A** | **p. P107Q** | **missense** | **24.2** | **Het** | **_** | **PD/**  **[29]** | **_** | **1,6** |
|  |  |  |  | ***PRKCG NM_002739*** | **Chr19:**  **54403680-54403680** | **c.1381 G>A** | **p. A461T** | **missense** | **26.3** | **Het** | **AD/**  **[30, 31]** | **SCAT1/**  **[32]** | **_** | **1,4,6** |
|  |  |  |  | ***EPHX1 NM_000120*** | **Chr1:**  **226026435-226026435** | **c.445 G>A** | **p. G149S** | **missense** | **36** | **Het** | **AD/**  **[33]** | **_** | **_** | **1,4** |
| **AD-18(F)** | **65** | **FMR** | **Ɛ3/ Ɛ4** | ***SERPINB1 NM_030666*** | **Chr6:**  **2833849-2833849** | **c.1133 C>A** | **p. S378Y** | **missense** | **27** | **Het** | ***AD/[34]** | **_** | **_** | **1,3** |
|  |  |  |  | ***RELN NM_005045*** | **Chr7:**  **103417007-103417007** | **c.541 G>C** | **p. G181R** | **missense** | **28.8** | **Het** | **AD/**  **[35]** | **ASD/**  **[35]** | **_** | **1,4,6** |
|  |  |  |  | ***GPR158 NM_020752*** | **Chr10:**  **25861798-25861798** | **c.1735 G>A** | **p. D579N** | **missense** | **34** | **Het** | **AD/**  **[36]** | **PD**  **[37]/**  **DP**  **[38]** | **_** | **1,4,6** |
|  |  |  |  | ***GOT1 NM_002079*** | **Chr10:**  **101180431-101180431** | **c.250 G>A** | **p. A84T** | **missense** | **20.8** | **Het** | **AD/**  **[39]** | **PD, HD**  **/[40]** | **_** | **1,4,6** |
|  |  |  |  | ***DIP2A NM_001146116*** | **Chr21:**  **47987294-47987294** | **c.4463 C>A** | **p. T1488N** | **missense** | **28.9** | **Het** | **AD/**  **[41]** | **DL, ASD/ [42]** | **_** | **1,4,6** |
|  |  |  |  | ***AIP NM_003977*** | **Chr11:**  **67256775-67256775** | **c.317 G>T** | **p. R106L** | **missense** | **25.7** | **Het** | ***AD/[43]** | **_** | **_** | **1,3** |
|  |  |  |  | ***FGFRL1 NM_001004356*** | **Chr4:**  **1018200-1018200** | **c.820 G>A** | **p. V274M** | **missense** | **29.6** | **Het** | **AD/**  **[44]** | **PD/**  **[44]** | **_** | **1,4,5** |
|  |  |  |  | ***ACTR10 NM_018477*** | **Chr14:**  **58701091-58701091** | **c.1076 C>T** | **p. A359V** | **missense** | **36** | **Het** | **AD/**  **[45]** | **_** | **_** | **1,4** |
|  |  |  |  | ***DVL2 NM_004422*** | **Chr17:**  **7131006-7131006** | **c.1199 G>A** | **p. S400N** | **missense** | **25.2** | **Het** | **AD/**  **[46]** | **_** | **_** | **1,4** |
|  |  |  |  | ***PAPPA NM_002581*** | **Chr9:**  **119124904-119124904** | **c.4381 G>A** | **p. D1461N** | **missense** | **27.7** | **Het** | **_** | **_** | **Ageing/ [47]** | **1,5** |
|  |  |  |  | ***EDEM2 NM_001145025*** | **Chr20:**  **33711716-33711716** | **c.980 G>A** | **p. R327Q** | **missense** | **25.5** | **Het** | **_** | **_** | **_** | **1** |
| **AD-20(M)** | **43** | **FMR** | **Ɛ3/ Ɛ3** | ***SYBU NM_001099749*** | **Chr8:**  **110590244-110590244** | **c.380 G>A** | **p. R127H** | **missense** | **25.3** | **Het** | **AD/**  **[48]** | **PD, LBD/ [48]** | **_** | **1,4,6** |
| **AD-23(F)** | **55** | **SP** | **Ɛ3/ Ɛ3** | ***PUM2 NM_001282752*** | **Chr2:**  **20494168-20494168** | **c.953 C>G** | **p. A318G** | **missense** | **21.2** | **Het** | **_** | **EP/**  **[49]** | **_** | **1,6** |
|  |  |  |  | ***BCAS1 NM_003657*** | **Chr20:**  **52612433-52612433** | **c.880 C>G** | **p. L294V** | **missense** | **20.7** | **Het** | **_** | **PD/**  **[50]** | **_** | **1,6** |
|  |  |  |  | ***ZNF544 NM_014480*** | **Chr19:**  **58772412-58772412** | **c.440 C>A** | **p. S147X** | **nonsense** | **35** | **Het** | **_** | **ADHD/**  **[51]** | **_** | **1,6** |
| **AD-25(M)** | **62** | **FMR** | **Ɛ3/ Ɛ3** | ***CASS4 NM_001164116*** | **Chr20:**  **55012575-55012575** | **c.392 A>G** | **p. Y131C** | **missense** | **23.9** | **Het** | **AD/**  **[52]** | **_** | **_** | **1,4** |
| **AD-26(M)** | **44** | **SP** | **Ɛ4/ Ɛ4** | ***CAPN1 NM_001198868*** | **Chr11:**  **64950266-64950266** | **c.94**  **G>T** | **p. E32X** | **nonsense** | **36** | **Het** | **AD/[53]** | **SP/**  **[54]** | **_** | **1,4,6** |
| **AD-32(F)** | **58** | **SP** | **Ɛ3/ Ɛ3** | ***N4BP3 NM_015111*** | **Chr5:**  **177548135- 177548135** | **c.889 C>G** | **p. L297V** | **nonsense** | **23.5** | **Het** | **_** | **_** | **Axonal and dendritic branching/**  **[55]** | **1,5** |
| **AD-34(F)** | **63** | **SP** | **Ɛ3/ Ɛ3** | ***PLK4 NM_001190801*** | **Chr4:**  **128804437-128804437** | **c.24 C>G** | **p.Y8X** | **nonsense** | **34** | **Het** | **_** | **PD/**  **[56]** | **_** | **1,6** |
| **AD-40(F)** | **73** | **FMR** | **Ɛ3/ Ɛ4** | ***SCARB2 NM_001204255*** | **Chr4:**  **77084506-77084506** | **c.841 C>T** | **p. R281X** | **nonsense** | **36** | **Het** | **_** | **LBD/**  **[57]** | **_** | **1,6** |
|  |  |  |  | ***SLC7A2 NM_001008539*** | **Chr8:**  **17409388-17409388** | **c.948 C>G** | **p. Y316X** | **nonsense** | **33** | **Het** | **AD/**  **[58]** | **_** | **_** | **1,4** |
| **AD-42(F)** | **84** | **FMR** | **Ɛ3/ Ɛ3** | ***MYH15 NM_014981*** | **Chr3:**  **108156426-108156426** | **c.3256 C>T** | **p. R1086X** | **nonsense** | **37** | **Het** | **_** | **ALS/**  **[59]** | **_** | **1,6** |
| **AD-43(F)** | **72** | **FMR** | **Ɛ3/ Ɛ4** | ***COL11A1 NM_080630*** | **Chr1:**  **103348801-103348801** | **c.4577 C>A** | **p. T1526K** | **missense** | **20.9** | **Het** | **AD/**  **[60]** | **_** |  | **1,4** |
|  |  |  |  | ***FANCC NM_000136*** | **Chr9:**  **97864072- 97864072** | **c.1594 A>G** | **p. R532G** | **missense** | **23.4** | **Het** | **AD**  **/[61]** | **PD/**  **[62]** | **_** | **1,4,6** |
|  |  |  |  | ***EPS8 NM_004447*** | **Chr12:**  **15803884-15803884** | **c.1307 G>T** | **p. G436V** | **missense** | **27.3** | **Het** | **AD/**  **[63]** | **_** | **_** | **1,4** |
|  |  |  |  | ***NFKBIE NM_004556*** | **Chr6:**  **44233331- 44233331** | **c.170 C>T** | **p. A57V** | **missense** | **25.4** | **Het** | **AD/**  **[64]** | **_** | **_** | **1,4** |
|  |  |  |  | ***HERPUD1 NM_001010989*** | **Chr16:**  **56970692-56970692** | **c.391 C>T** | **p. R131W** | **missense** | **26.4** | **Het** | **_** | **_** | **Atherosclerosis**  **/[65]** | **1,5** |
| **AD-45(M)** | **70** | **FMR** | **Ɛ4/ Ɛ4** | ***TCF7L1 NM_031283*** | **Chr2:**  **85533441-85533441** | **c.1102 T>G** | **p. C368G** | **missense** | **20.1** | **Het** | **AD/**  **[66]** | **_** |  | **1,4** |
|  |  |  |  | ***DGKD NM_003648*** | **Chr2:**  **234343503-234343503** | **c.410 G>A** | **p. R137H** | **missense** | **24.3** | **Het** | **_** | **EP/**  **[67]** | **_** | **1,6** |
|  |  |  |  | ***AMOTL1 NM_001301007*** | **Chr11:**  **94532922-94532922** | **c.416 C>T** | **p. P139L** | **missense** | **20.7** | **Het** | **AD/**  **[68]** | **_** | **_** | **1,4** |
| **AD-48(F)** | **85** | **FMR** | **Ɛ3/ Ɛ3** | ***PDK1 NM_002610*** | **Chr16:**  **173457666-173457666** | **c.1060 G>T** | **p. G354C** | **missense** | **27.4** | **Het** | ***AD/[69]** | **_** | **_** | **1,3,4** |
| **AD-49(F)** | **84** | **FMR** | **Ɛ3/ Ɛ4** | ***ARHGEF38 NM_001242729*** | **Chr4:**  **106588368-106588368** | **c.1772 G>A** | **p. R591H** | **missense** | **32** | **Het** | **_** | **EP/**  **[70]** | **_** | **1,6** |
|  |  |  |  | ***SLC1A3***  ***NM_001289940*** | **Chr5:**  **36686192-36686192** | **c.1114 G>A** | **p. V372I** | **missense** | **36** | **Het** | **AD/ [71]** | **EAs/**  **[72]** | **_** | **1,4,6** |
|  |  |  |  | ***CACNA2D4 NM_172364*** | **Chr12:**  **1995135-1995135** | **c.1064 G>A** | **p. R355Q** | **missense** | **34** | **Het** | **AD/**  **[73, 74]** | **_** | **_** | **1,4** |
| **AD-50(F)** | **75** | **FMR** | **Ɛ3/ Ɛ3** | ***MBOAT2 NM_138799*** | **Chr2:**  **9002724-9002724** | **c.1181 G>A** | **p. R394K** | **missense** | **34** | **Het** | **_** | **MS/**  **[75]** | **_** | **1,6** |
|  |  |  |  | ***ABLIM1 NM_006720*** | **Chr10:**  **116232819-116232819** | **c.328 A>C** | **p. I 110L** | **missense** | **26** | **Het** | **_** | **ALS, FTD/**  **[76]** | **_** | **1,6** |
|  |  |  |  | ***QPRT NM_014298*** | **Chr16:**  **29706284-29706284** | **c.313 C>G** | **p. L105V** | **missense** | **23.1** | **Het** | ***AD/[77]** | **_** | **_** | **1,3** |
|  |  |  |  | ***ABCA8 NM_007168*** | **Chr17:**  **66871833-66871833** | **c.4292 C>A** | **p. A1431D** | **missense** | **25.5** | **Het** | **_** | **MSA/**  **[78]** | **_** | **1,6** |
|  |  |  |  | ***RADIL NM_018059*** | **Chr7:**  **4871776-4871776** | **c.1450 C>T** | **p. Q484X** | **nonsense** | **40** | **Het** | **_** | **_** | **Regulator of neural crest migration/**  **[79]** | **1,6** |
| **AD-53(F)** | **77** | **FMR** | **Ɛ3/ Ɛ4** | ***HMGCLL1***  ***NM_001287753*** | **Chr6:**  **55304296-55304296** | **c.548 G>A** | **p. G183D** | **missense** | **33** | **Het** | **_** | **SCZ/**  **[80]** |  | **1,6** |
|  |  |  |  | ***TTC39C NM_001135993*** | **Chr18:**  **21662948-21662948** | **c.887 G>A** | **p. G296D** | **missense** | **30** | **Het** | **AD/**  **[81]** | **EP/**  **[82]** |  | **1,4,6** |
|  |  |  |  | ***TRIM66 NM_014818*** | **Chr11:**  **8643323-8643323** | **c.2596 C>T** | **p. R866X** | **nonsense** | **43** | **Het** | **_** | **MS/**  **[83]** | **_** | **1,6** |
| **AD-54(F)** | **69** | **FMR** | **Ɛ3/ Ɛ4** | ***TNR NM_003285*** | **Chr1:**  **175360456-175360456** | **c.1475 G>A** | **p. R492H** | **missense** | **33** | **Het** | **AD/**  **[60]** | **DD/**  **[84]** | **_** | **1,4,6** |
|  |  |  |  | ***ITPKA NM_002220*** | **Chr15:**  **41795245-41795245** | **c.1267 C>A** | **p. L423I** | **missense** | **27.5** | **Het** | **_** | **HD/**  **[85]** | **_** | **1,6** |
|  |  |  |  | ***DHX34 NM_014681*** | **Chr19:**  **47878865-47878865** | **c.2207 G>A** | **p. R736H** | **missense** | **36** | **Het** | **_** | **DD, ID/**  **[86]** | **_** | **1,6** |
|  |  |  |  | ***SACS NM_014363*** | **Chr13:**  **23908126-23908126** | **c.9889 G>T** | **p. E3297X** | **nonsense** | **58** | **Het** | **_** | **EP, ID/**  **[87]** | **_** | **1,6** |
| **AD-55(F)** | **71** | **FMR** | **Ɛ3/ Ɛ3** | ***PTPN9 NM_002833*** | **Chr15:**  **75798281-53928395** | **c.703 C>A** | **p. L235M** | **missense** | **23.2** | **Het** | **AD/**  **[88]** | **_** | **_** | **1,4** |
|  |  |  |  | ***ATF7 NM_006856*** | **Chr12:**  **53928395-53928395** | **c.451 G>A** | **p. V151I** | **missense** | **26.9** | **Het** | **AD/**  **[89]** | **_** | **_** | **1,4,6** |
|  |  |  |  | ***LIG3 NM_013975*** | **Chr17:**  **33321338-33321338** | **c.1499 C>T** | **p. P500L** | **missense** | **33** | **Het** | **AD/**  **[90]** | **AT/**  **[91]** | **_** | **1,4,6** |
|  |  |  |  | ***ACSS2 NM_018677*** | **Chr20:**  **33470747-33470747** | **c.329 G>A** | **p. R110Q** | **missense** | **35** | **Het** | **_** | **_** | **Memory/**  **[92]** | **1,5** |
| **AD-56(M)** | **66** | **SP** | **Ɛ3/ Ɛ4** | ***SERINC2 NM_018565*** | **Chr1:**  **31905938-31905938** | **c.1150 G>A** | **p. E384K** | **missense** | **34** | **Het** | **_** | **ASD/**  **[93]** |  | **1,6** |
|  |  |  |  | ***∞FLNC NM_001458*** | **Chr7:**  **128481592- 128481592** | **c.2092 G>A** | **p. G698S** | **missense** | **37** | **Het** | **AD/**  **[94]** | **FTD/**  **[95]** | **_** | **1,4,6** |
|  |  |  |  | ***HERC1 NM_003922*** | **Chr15:**  **64046796- 64046796** | **c.1682 G>A** | **p. S561N** | **missense** | **24.8** | **Het** | **_** | **ASD/**  **[96]** | **_** | **1,6** |
|  |  |  |  | ***PSMF1 NM_006814*** | **Chr20:**  **1106213-1106213** | **c.202 C>T** | **p. R68W** | **missense** | **23.6** | **Hom** | **AD/**  **[97]** | **_** | **_** | **1,4** |
|  |  |  |  | ***MEP1B NM_005925*** | **Chr18:**  **29784230-29784230** | **c.454 C>T** | **p. H152Y** | **missense** | **27.1** | **Het** | ***AD/[98]** | **_** | **_** | **1,3** |
| **AD-59(M)** | **68** | **SP** | **Ɛ3/ Ɛ3** | ***SLC30A7 NM_001144884*** | **Chr1:**  **101376707-101376707** | **c.384+1 G>T** | **_** | **splice site** | **29** | **Het** | ***AD/[99]** | **MS/**  **[100]** | **_** | **1,3,6** |
|  |  |  |  | ***CRYM NM_001888*** | **Chr16:**  **21272589-21272589** | **c.865 _/C** | **p. V289fs** | **frameshift** | **25.1** | **Hom** | **AD/**  **[101]** | **_** | **_** | **1,4** |
|  |  |  |  | ***GRK5 NM_005308*** | **Chr10:**  **121203179-121203179** | **c.1181 G>A** | **p. R394Q** | **missense** | **26.8** | **Het** | ***AD/**  **[102]** | **_** | **_** | **1,3** |
| **AD-60(M)** | **79** | **SP** | **Ɛ3/ Ɛ3** | ***KALRN***  ***NM_007064*** | **Chr3:**  **124385404-124385404** | **c.1360 G>C** | **p. E454Q** | **missense** | **21.5** | **Het** | **AD/**  **[103]** | **_** | **_** | **1,4** |
|  |  |  |  | ***AXIN2 NM_004655*** | **Chr17:**  **63533805-63533805** | **c.1349 C>T** | **p. P450L** | **missense** | **25.3** | **Het** | **AD/**  **[104]** | **DS/**  **[104]** | **_** | **1,4,6** |
|  |  |  |  | ***MAPKAP1 NM_001006620*** | **Chr9:**  **128201281-128201281** | **c.878 T>A** | **p. I293N** | **missense** | **29.3** | **Het** | **_** | **FMD/**  **[105]** | **_** | **1,6** |
|  |  |  |  | ***TIMELESS NM_003920*** | **Chr12:**  **56814611-56814611** | **c.3095 A>G** | **p. D1032G** | **missense** | **23.4** | **Het** | **AD/**  **[106]** | **BPD/**  **[106]** | **_** | **1,4,6** |
|  |  |  |  | ***PTPRH NM_001161440*** | **Chr19:**  **55697642-55697642** | **c.2195 A>G** | **p. Q732R** | **missense** | **22.6** | **Het** | **_** | **PD/**  **[107]** | **_** | **1,6** |
| **AD-70(F)** | **67** | **SP** | **Ɛ3/ Ɛ4** | ***DAB1 NM_021080*** | **Chr1:**  **57538005-57538005** | **c.389 G>A** | **p.C130Y** | **missense** | **21.2** | **Het** | **AD/**  **[60]** | **_** | **_** | **1,4,6** |
|  |  |  |  | ***LAP3; LAP3 NM_015907*** | **Chr4:**  **17600095-17600095** | **c.1094 C>G** | **p. A365G** | **missense** | **29.8** | **Het** | **AD/**  **[108]** | **_** |  | **1,4** |
|  |  |  |  | ***JADE2 NM_015288*** | **Chr5:**  **133871607-133871607** | **c.58+2 T>C** | **_** | **splice site** | **20** | **Het** | **_** | **_** | **Synaptic Plasticity and Cognitive Functions**  **/[109]** | **1,5** |
| **AD-73(M)** | **75** | **SP** | **Ɛ3/ Ɛ3** | ***SUPT4H1; BZRAP1-AS1 NM_003168*** | **Chr17:**  **56423651-56423651** | **c.310 C>T** | **p. R104X** | **nonsense** | **37** | **Het** | **_** | **ALS, FTD/ [110]** | **_** | **1,6** |
| **AD-76(F)** | **80** | **SP** | **Ɛ3/ Ɛ4** | ***UNC80 NM_032504*** | **Chr2:**  **210841707-210841707** | **c.8645 G>A** | **p. R2882Q** | **missense** | **26.7** | **Het** | **AD/ [111]** | **BPD, ASD, EP, SCZ**  **/[111]** | **_** | **1,4,6** |
|  |  |  |  | ***DOCK4 NM_014705*** | **Chr7:**  **111430661-111430661** | **c.3167 G>T** | **p. G1056V** | **missense** | **24.6** | **Het** | **_** | **ASD/**  **[112]** | **_** | **1,6** |
|  |  |  |  | ***JADE1 (PHF17)***  ***NM_001287437*** | **Chr4:**  **129770232-129770232** | **c.358 C>T** | **p. R120W** | **missense** | **22.8** | **Het** | **_** | **PART/**  **[113]** | **_** | **1,6** |
| **AD-78(M)** | **76** | **SP** | **Ɛ3/ Ɛ4** | ***WDFY3 NM_014991*** | **Chr4:**  **85603544-85603544** | **c.9806 G>A** | **p.C3269Y** | **missense** | **21.5** | **Het** | ***AD/[114]** | **DD/**  **[115]** | **_** | **1,4,6** |
|  |  |  |  | ***SHC3 NM_016848*** | **Chr9:**  **91653003-91653003** | **c.1561 G>A** | **p. V521I** | **missense** | **33** | **Het** | ***AD/[116]** | **_** | **_** | **1,4,6** |
|  |  |  |  | ***SLC6A15 NM_001146335*** | **Chr12:**  **85267071-85267071** | **c.583 G>T** | **p. A195S** | **missense** | **35** | **Het** | **AD/**  **[117]** | **MDD/**  **[117]** | **_** | **1,4,6** |
|  |  |  |  | ***AOX1 NM_001159*** | **Chr2:**  **201521638-201521638** | **c.3149 G>A** | **p. G1050E** | **missense** | **27.3** | **Het** | **AD/**  **[118]** | **_** | **_** | **1,3,4** |
|  |  |  |  | ***KLF11 NM_001177718*** | **Chr2:**  **10188668-10188668** | **c.1153 C>T** | **p. R385W** | **missense** | **20.4** | **Het** | **_** | **IS/**  **[119]** | **_** | **1,6** |
| **AD-80(F)** | **83** | **SP** | **Ɛ3/ Ɛ3** | ***PDE4DIP NM_001002811*** | **Chr1:144915561-144915561** | **c.2353 C>T** | **p. R785X** | **nonsense** | **40** | **Het** | **AD/**  **[9]** | **FTD, VaD/**  **[10]** | **_** | **1,2,4,6** |
| **AD-83(M)** | **61** | **SP** | **Ɛ3/ Ɛ3** | ***DOCK10***  ***NM_001290263*** | **Chr2:**  **225651822-225651822** | **c.5554 C>T** | **p. R1852W** | **missense** | **31** | **Het** | **_** | **PD/**  **[4]** | **_** | **1,6** |
|  |  |  |  | ***PON2 NM_000305*** | **Chr7:**  **95041743-95041743** | **c.248 G>A** | **p. G83E** | **missense** | **26.1** | **Het** | **AD/**  **[120]** | **_** | **_** | **1,4,6** |
|  |  |  |  | ***CLOCK NM_001267843*** | **Chr4:**  **56345042-56345042** | **c.196 A>G** | **p. R66G** | **missense** | **21.1** | **Het** | ***AD/[121]** | **ASD/ [122], PD/ [123]** | **_** | **1,4,6** |
| **AD-84(F)** | **56** | **FMR** | **Ɛ3/ Ɛ4** | ***TMEM232 NM_001039763*** | **Chr5:**  **109904269-109904269** | **c.1334 C>A** | **p. S445X** | **nonsense** | **28.8** | **Het** | **AD/**  **[124]** | **_** | **_** | **1,4** |
|  |  |  |  | ***BMP3 NM_001201*** | **Chr4:**  **81967149-81967149** | **c.574 C>T** | **p. R192X** | **nonsense** | **20.5** |  | **_** | **_** | **Integrity of Blood Brain Barrier/[125]** | **1,5** |

**Keys:** AAO, Age at onset; #P, Number of prioritization criteria; Het, Heterozygous; Hom, Homozygous; PD, Parkinson's Disease; MS, Multiple Sclerosis; RDD, Recurrent Depressive Disorder; EP, Epilepsy; CD, Cognitive Dysfunction; FTD, Frontotemporal dementia; VaD, Vascular dementia; PNK, Paroxysmal nonkinesigenic dyskinesia; LBD, Lewy body dementia; FP, Familial Psychosis; HD, Huntington's disease; SCAT14, Spinocerebellar Ataxia Type 14; ASD, Autism; AT, Ataxia Telangiectasia; DL, Dyslexia; ALS, Amyotrophic lateral sclerosis; SP, Spastic Paraplegia; EAs, Episodic ataxias; MSA, Multiple system atrophy; DD, Developmental delay; ID, Intellectual disability; FMD, Familial mood disorder; BPD, Bipolar disorder; SCZ, Schizophrenia; ADHD, Attention-deficit/hyperactivity disorder; DS, Down syndrome; PART, Primary age-related tauopathy; IS, Ischemic stroke. * Indicates variants involved in the metabolism of amyloid-β (Aβ) peptide or Tau proteins.

**S3 Table: Rare candidate variants identified in the AD cohort with ClinVar annotation and ACMG/AMP classification.**

| **Proband ID/Gender** | **Variant Identification** | | | | **Population Frequency** | **Reporting Status** | | **Interpretation** | |
| --- | --- | --- | --- | --- | --- | --- | --- | --- | --- |
|  | **Gene** | **Transcript** | **Protein** | **rsID** | **gnomAD Frequency** | **ClinVar** | **Reported/**  **Novel** | **ACMG Classification** | **ACMG Evidence** |
| AD-8(F) | DOCK8 | NM_001190458 | p.V898I | rs751201571 | 0.00000164 | VUS | Reported | VUS | PM2, BP4 |
|  | FKBP5 | NM_001145777 | p.P246L | rs180672585 | 0.0000526 | Not reported | Reported | VUS | PM2, BP4 |
|  | MAN1C1 | NM_020379 | - | Not available | Not observed | Not reported | Novel | Likely Pathogenic | PVS1, PM2 |
| AD-10(M) | MMP2 | NM_001127891 | p.S102G | Not available | Not observed | Not reported | Novel | VUS | PM2, BP4 |
| AD-11(F) | CALHM1 | NM_001001412 | p.E233X | rs573376053 | Not observed | Not reported | Reported | Likely Pathogenic | PVS1, PM2 |
|  | GLP2R | NM_004246 | p.S5X | Not available | Not observed | Not reported | Novel | Likely Pathogenic | PVS1, PM2 |
|  | PDE4DIP | NM_001395426 | p.Q454E | rs587602816 | 0.00000345 | Not reported | Reported | VUS | PM2, BP4 |
| AD-13(F) | NBAS | NM_015909 | p.W1409X | rs2528426837 | Not observed | Pathogenic | Reported | Pathogenic | PVS1, PM2 |
| AD-14(F) | CD36 | NM_001289908 | p.A419D | rs866158583 | Not observed | Not reported | Reported | VUS | PM2, PP3 |
|  | DHX57 | NM_198963 | p.V1340M | rs868327057 | 0.00000275 | Not reported | Reported | VUS | PM2, PP3 |
|  | LIMK1 | NM_001204426 | p.V512M | Not available | Not observed | Not reported | Novel | VUS | PM2, PP3 |
| AD-16(F) | KCNMA1 | NM_001014797 | p.S59F | rs2552275366 | 0.00000548 | VUS | Reported | VUS | PM2, BP4 |
|  | PLCG2 | NM_002661 | p.R461X | rs778484571 | 0.0000595 | VUS | Reported | VUS | PM2 |
|  | PTDSS1 | NM_014754 | p.T304S | rs1586200351 | 0.0000263 | VUS | Reported | VUS | PM2, PP3 |
| AD-17(M) | SLC17A6 | NM_020346 | p.I427V | rs747226260 | 0.00000657 | Not reported | Reported | VUS | PM2 |
|  | STEAP4 | NM_024636 | p.R50Q | rs750091751 | 0.0000616 | Not reported | Reported | VUS | PM2, PP3 |
|  | ADAMTS8 | NM_007037 | p.G384R | rs70656228 | 0.0000575 | Not reported | Reported | VUS | PM2, PP3 |
|  | TMEM87A | NM_001286487 | p.Q406E | Not available | Not observed | Not reported | Novel | VUS | PM2 |
|  | RASL12 | NM_016563 | p.Y98H | rs929043708 | Not observed | Not reported | Reported | VUS | PM2, PP3 |
|  | MACF1 | NM_012090 | p.P125A | rs1645074548 | Not observed | VUS | Reported | VUS | PM2, PP3 |
|  | ASTN1 | NM_004319 | p.R1270W | rs12118933 | 0.0000395 | Not reported | Reported | VUS | PM2, PP3 |
|  | FH | NM_000143 | p.R350Q | rs749316923 | 0.0000219 | VUS | Reported | VUS | PM2, PP3 |
|  | TCERG1 | NM_006706 | p.R552H | rs779452497 | 0.00000891 | Not reported | Reported | VUS | PM2, PP3 |
|  | LONP1 | NM_004793 | p.P107Q | rs748759945 | 0.00000959 | VUS | Reported | Likely Benign | BS1, BP4 |
|  | PRKCG | NM_002739 | p.A461T | rs749266717 | 0.0000752 | Likely Pathogenic | Reported | Likely Pathogenic | PM2, PP3 |
|  | EPHX1 | NM_000120 | p.G149S | rs769650380 | 0.0000253 | Not reported | Reported | VUS | PM2 |
| AD-18(F) | SERPINB1 | NM_030666 | p.S378Y | rs764085764 | 0.00000413 | Not reported | Reported | VUS | PM2 |
|  | RELN | NM_005045 | p.G181R | rs1342417375 | 0.00000342 | Not reported | Reported | VUS | PM2, PP3 |
|  | GPR158 | NM_020752 | p.D579N | Not available | Not observed | Not reported | Novel | VUS | PM2 |
|  | GOT1 | NM_002079 | p.A84T | Not available | Not observed | Not reported | Novel | VUS | PM2 |
|  | DIP2A | NM_001146116 | p.T1488N | Not available | Not observed | Not reported | Novel | VUS | PM2 |
|  | AIP | NM_003977 | p.R106L | rs373950586 | 0.000000685 | VUS | Reported | VUS | PM2, PP3 |
|  | FGFRL1 | NM_001004356 | p.V274M | rs754703776 | 0.0000131 | VUS | Reported | VUS | PM2, BP4 |
|  | ACTR10 | NM_018477 | p.A359V | rs756285067 | 0.0000489 | VUS | Reported | VUS | PM2, BP4 |
|  | DVL2 | NM_004422 | p.S400N | rs760962550 | 0.0000328 | VUS | Reported | VUS | PM2, BP4 |
|  | PAPPA | NM_002581 | p.D1461N | Not available | Not observed | Not reported | Novel | VUS | PM2, BP4 |
|  | EDEM2 | NM_001145025 | p.R327Q | rs753615040 | 0.0000251 | VUS | Reported | VUS | PM2 |
| AD-20(M) | SYBU | NM_001099749 | p.R127H | rs575944059 | 0.0000296 | Not reported | Reported | VUS | PM2 |
| AD-23(F) | PUM2 | NM_001282752 | p.A318G | rs2529602131 | 0.00000684 | Not reported | Reported | VUS | PM2 |
|  | BCAS1 | NM_003657 | p.L294V | rs1432944349 | 0.00000692 | Not reported | Reported | VUS | PM2 |
|  | ZNF544 | NM_014480 | p.S147X | rs753737901 | 0.0000103 | Not reported | Reported | Likely pathogenic | PVS1, PM2 |
| AD-25(M) | CASS4 | NM_001164116 | p.Y131C | Not available | 0.000016 | Not reported | Reported | VUS | PM2, PP3 |
| AD-26(M) | CAPN1 | NM_001198868 | p.E32X | Not available | Not observed | Not reported | Novel | Likely pathogenic | PVS1, PM2 |
| AD-32(F) | N4BP3 | NM_015111 | p.L297V | Not available | Not observed | Not reported | Novel | VUS | PM2, PP3 |
| AD-34(F) | PLK4 | NM_001190801 | p.Y8X | Not available | Not observed | Not reported | Novel | Likely pathogenic | PVS1, PM2 |
| AD-40(F) | SCARB2 | NM_001204255 | p.R281X | rs886041078 | 0.00000684 | Not reported | Reported | Pathogenic | PVS1, PM2 |
|  | SLC7A2 | NM_001008539 | p.Y316X | rs2486527240 | 0.00000684 | Not reported | Reported | Likely pathogenic | PVS1, PM2 |
| AD-42(F) | MYH15 | NM_014981 | p.V1086L | Not available | Not observed | Not reported | Novel | VUS | PM2, PP3 |
| AD-43(F) | COL11A1 | NM_080630 | p.T1526K | rs1173031118 | 0.0000137 | Not reported | Reported | VUS | PM2, BP4 |
|  | FANCC | NM_000136.3 | p.R532G | rs764649916 | 0.00000684 | Not reported | Reported | VUS | PM2, PP3 |
|  | EPS8 | NM_004447.6 | p.G436V | rs555365153 | 0.0000131 | Not reported | Reported | VUS | PM2, PP3 |
|  | NFKBIE | NM_004556.3 | p.E57V | Not available | Not observed | Not reported | Novel | VUS | PM2, PP3 |
|  | HERPUD1 | NM_001010989.3 | p.R131W | rs527424441 | 0.000855 | Not reported | Reported | VUS | PM2 |
| AD-45(M) | TCF7L1 | NM_031283.3 | p.C368G | rs1682106512 | Not observed | Not reported | Reported | VUS | PM2 |
|  | DGKD | NM_003648.3 | p.R137H | rs779966904 | 0.0000869 | VUS | Reported | VUS | PM2 |
|  | AMOTL1 | NM_001301007.2 | p.P139L | rs1951435731 | 0.000000686 | Not reported | Reported | VUS | PM2 |
| AD-48(F) | PDK1 | NM_002610.5 | p.G354C | rs2468947153 | 0.000000694 | Not reported | Reported | VUS | PM2 |
| AD-49(F) | ARHGEF38 | NM_001242729.2 | p.R591H | rs939877535 | 0.0000131 | Not reported | Reported | VUS | PM2 |
|  | SLC1A3 | NM_001289940.2 | p.V372I | rs549164007 | 0.0000212 | VUS | Reported | VUS | PM2 |
|  | CACNA2D4 | NM_172364.5 | p.R355Q | rs781209342 | 0.0000329 | VUS | Reported | VUS | PM2 |
| AD-50(F) | MBOAT2 | NM_138799.4 | p.R394K | rs2528637247 | 0.000000687 | Not reported | Reported | VUS | PM2 |
|  | ABLIM1 | NM_006720.4 | p.I110L | rs1343862747 | 0.00000137 | Not reported | Reported | VUS | PM2 |
|  | QPRT | NM_014298.6 | p.L105V | Not available | Not observed | Not reported | Novel | VUS | PM2, BP4 |
|  | ABCA8 | NM_007168.4 | p.A1431D | Not available | Not observed | Not reported | Novel | VUS | PM2, BP4 |
|  | RADIL | NM_018059.5 | p.Q484X | rs533522927 | 0.0000591 | Not reported | Reported | VUS | PVS1, PM2 |
| AD-53(F) | HMGCLL1 | NM_001287753.2 | p.G183D | rs1763505957 | 0.00000685 | Not reported | Reported | VUS | PM2, PP3 |
|  | TTC39C | NM_001135993.2 | p.G296D | rs2511856206 | 0.00000137 | VUS | Reported | VUS | PM2, PP3 |
|  | TRIM66 | NM_014818.2 | p.R866X | rs1350056057 | 0.00002 | Not reported | Reported | VUS | PVS1, PM2 |
| AD-54(F) | TNR | NM_003285.3 | p.R492H | rs984032476 | 0.00000753 | Not reported | Reported | VUS | PM2, PP3 |
|  | ITPKA | NM_002220.3 | p.L423I | Not available | Not observed | Not reported | Novel | VUS | PM2, BP4 |
|  | DHX34 | NM_014681.6 | p.R736H | rs1316446900 | 0.0000043 | Not reported | Reported | VUS | PM2, PP3 |
|  | SACS | NM_014363.6 | p.E3297X | Not available | Not observed | Not reported | Novel | VUS | PVS1, PM2 |
| AD-55(F) | PTPN9 | NM_002833.4 | p.L235M | Not available | Not observed | Not reported | Novel | VUS | PM2, PP3 |
|  | ATF7 | NM_006856.3 | p.V151I | rs1237825732 | 0.0000137 | Not reported | Reported | VUS | PM2, PP3 |
|  | LIG3 | NM_013975.4 | p.P500L | rs2508958709 | 0.00000684 | Not reported | Reported | VUS | PM2, PP3 |
|  | ACSS2 | NM_018677.4 | p.R110Q | rs757812388 | 0.000185 | Not reported | Reported | VUS | PM2, PP3 |
| AD-56(M) | SERINC2 | NM_018565.4 | p.E384K | rs782127025 | 0.000986 | Not reported | Reported | VUS | PM2, PP3 |
|  | FLNC | NM_001458.5 | p.G698S | Not available | 0.000013 | VUS | Reported | VUS | PM2, PP3 |
|  | HERC1 | NM_003922.4 | p.S561N | rs2551772968 | 0.00000684 | Not reported | Reported | VUS | PM2, PP3 |
|  | PSMF1 | NM_006814.5 | p.R68W | rs775760080 | 0.000394 | Not reported | Reported | VUS | PM2, PP3 |
|  | MEP1B | NM_005925.3 | p.H152Y | rs780845126 | 0.00000757 | Not reported | Reported | VUS | PM2, BP4 |
| AD-59(M) | SLC30A7 | NM_001144884 | - | Not available | Not observed | Not reported | Novel | VUS | PM2, PP3 |
|  | CRYM | NM_001888.5 | p.V289fs | Not available | Not observed | Not reported | Novel | VUS | PM2, PP3 |
|  | GRK5 | NM_005308.3 | p.R394Q | rs750739113 | 0.0000263 | Not reported | Reported | VUS | PM2, PP3 |
| AD-60(M) | KALRN | NM_007064.5 | p.E454Q | rs767728290 | 0.00000684 | Not reported | Reported | VUS | PM2, PP3 |
|  | AXIN2 | NM_004655.4 | p.P450L | rs777265234 | 0.00000498 | Not reported | Reported | VUS | PM2, PP3 |
|  | MAPKAP1 | NM_001006620.2 | p.I293N | rs1467551848 | 0.00000205 | Not reported | Reported | VUS | PM2, PP3 |
|  | TIMELESS | NM_003920.5 | p.D1032G | rs746185610 | 0.00000547 | Not reported | Reported | VUS | PM2, BP4 |
|  | PTPRH | NM_001161440.3 | p.Q732R | rs779339619 | 0.00000958 | Not reported | Reported | VUS | PM2, BP4 |
| AD-70(F) | DAB1 | NM_021080.5 | p.C130Y | rs1651560169 | Not observed | Not reported | Reported | VUS | PM2, PP3 |
|  | LAP3 | NM_015907.3 | p.A365G | Not available | Not observed | Not reported | Novel | VUS | PM2, BP4 |
|  | JADE2 | NM_015288.6 | - | Not available | Not observed | Not reported | Novel | VUS | PM2 |
| AD-73(M) | SUPT4H1 | NM_003168.3 | p.R104X | rs1972284656 | 0.00000616 | Not reported | Reported | VUS | PM2 |
| AD-76(F) | UNC80 | NM_032504.2 | p.R2882Q | rs535321786 | 0.0000131 | VUS | Reported | VUS | PM2 |
|  | DOCK4 | NM_014705.4 | p.G1056V | Not available | Not observed | Not reported | Novel | VUS | PM2, PP3 |
|  | JADE1 | NM_001287437.2 | p.R120W | rs1400139623 | 0.00000752 | Not reported | Reported | VUS | PM2, BP4 |
| AD-78(M) | WDFY3 | NM_014991.6 | p.C3269Y | rs780379413 | 0.0000131 | VUS | Reported | VUS | PM2 |
|  | SHC3 | NM_016848.6 | p.V521I | rs755281337 | 0.0000301 | VUS | Reported | VUS | PM2 |
|  | SLC6A15 | NM_001146335.3 | p.A195S | rs1263416284 | 0.000000684 | Not reported | Reported | VUS | PM2, PP3 |
|  | AOX1 | NM_001159.4 | p.G1050E | Not available | Not observed | Not reported | Novel | VUS | PM2, PP3 |
|  | KLF11 | NM_001177718.2 | p.R385W | rs545920064 | 0.0000525 | VUS | Reported | VUS | PM2, PP3 |
| AD-80(F) | PDE4DIP | NM_001002811.3 | p.R785X | rs1778111 | 0.000000684 | Not reported | Reported | Likely Pathogenic | PVS1, PM2 |
| AD-83(M) | DOCK10 | NM_001290263.2 | p.R1852W | rs777865939 | 0.00000958 | Not reported | Reported | VUS | PM2, PP3 |
|  | PON2 | NM_000305.3 | p.G83E | rs553703460 | 0.000046 | VUS | Reported | VUS | PM2, PP3 |
|  | CLOCK | NM_001267843.2 | p.R66G | Not available | Not observed | Not reported | Novel | VUS | PM2, PP3 |
| AD-84(F) | TMEM232 | NM_001039763.4 | p.S445X | rs1177658274 | 0.0000132 | Not reported | Reported | Likely Pathogenic | PVS1, PM2 |
|  | BMP3 | NM_001201.5 | p.R192X | rs759472288 | 0.00000657 | Not reported | Reported | Likely Pathogenic | PVS1, PM2 |

Keys: ACMG/AMP evidence codes: PVS1 (predicted loss-of-function variant in a gene with an established disease mechanism), PM2 (absent or extremely rare in control populations), PP3 (multiple lines of computational evidence supporting a deleterious effect), BP4 (multiple lines of computational evidence supporting a benign effect). VUS, variants of uncertain significance.

**Author Contributions**

Fadia El Bitar (Conceptualization; Supervision and management of the present research; Experimental design; Analysis and interpretation of results and writing manuscript); Ghadeer Al Dawsari (Preparing all the tables in the manuscript; Participating in variants selection and sequence validation of the candidate variants); Najeeb Qadi (Patient recruitment and assessment); Saad Al Rajeh (Patient recruitment and assessment); Mohamed Abouelhoda (Supervision of WES bioinformatics analysis); Sahar Al Subaie (Participating in variants selection and sequence validation of the candidate variants); Amna Magrashi (Participating in sequence validation of the candidate variants); Hala Al Amari (Participating in sequence validation of the candidate variants); Fatimah Alghamdi (Participating in sequence validation of the candidate variants); Sara Abdulaziz (Receiving and processing blood samples, ensuring the presence of complete consent forms and filing of all the documents related to patient’s information and the family history); Bashayer Al Mubarak (Critical review of the manuscript). Nada Al Tassan (Analysis and interpretation of results; Critical review of the manuscript).

**Funding**

The authors have no funding to report.

**Institutional Review Board Statement**

Neurologists reviewed all patients, and their complete clinical and family histories were recorded. The study was conducted in accordance with the Declaration of Helsinki, with written informed consents obtained after receiving approval from the Institutional Review Board (IRB) at King Faisal Specialist Hospital and Research Center (RAC#2120020).

**Informed consent statement**

Written informed consent was obtained from all participants following the approval of the study by the Institutional Review Board (IRB) at King Faisal Specialist Hospital and Research Center (RAC#2120020).

**Data Availability**

The data supporting the findings of this study are available within the article and/or its supplementary material.

**Acknowledgements**

We deeply honor and remember Dr. Saad Al Rajeh, Consultant Neurologist, who passed away before seeing this research published. We are profoundly grateful for his pioneering work, dedication to science, collaboration, and profound insights. This publication is dedicated to his memory.

We would like to extend our thanks to Mr. Abdullah AlAhmad from Neuroscience Center of Excellence at KFSHRC and Head of main laboratory Mr. Oudah Alhamad at Al Habib Medical Group (Olaya Branch) for their assistance in blood collection from Alzheimer’s patients.

**Conflict of Interest**

The authors have no conflict of interest to report.

**References**

1. Namekata K, Guo X, Kimura A, Arai N, Harada C, Harada T. DOCK8 is expressed in microglia, and it regulates microglial activity during neurodegeneration in murine disease models. The Journal of biological chemistry. 2019;294(36):13421-33.

2. Bailus BJ, Scheeler SM, Simons J, Sanchez MA, Tshilenge KT, Creus-Muncunill J, et al. Modulating FKBP5/FKBP51 and autophagy lowers HTT (huntingtin) levels. Autophagy. 2021;17(12):4119-40.

3. Mikolas P, Tozzi L, Doolin K, Farrell C, O'Keane V, Frodl T. Effects of early life adversity and FKBP5 genotype on hippocampal subfields volume in major depression. J Affect Disord. 2019;252:152-9.

4. Calligaris R, Banica M, Roncaglia P, Robotti E, Finaurini S, Vlachouli C, et al. Blood transcriptomics of drug-naïve sporadic Parkinson's disease patients. BMC genomics. 2015;16:876.

5. Hernandez-Guillamon M, Mawhirt S, Blais S, Montaner J, Neubert TA, Rostagno A, et al. Sequential Amyloid-β Degradation by the Matrix Metalloproteases MMP-2 and MMP-9. The Journal of biological chemistry. 2015;290(24):15078-91.

6. Bobińska K, Szemraj J, Czarny P, Gałecki P. Role of MMP-2, MMP-7, MMP-9 and TIMP-2 in the development of recurrent depressive disorder. J Affect Disord. 2016;205:119-29.

7. Louis S, Busch RM, Lal D, Hockings J, Hogue O, Morita-Sherman M, et al. Genetic and molecular features of seizure-freedom following surgical resections for focal epilepsy: A pilot study. Front Neurol. 2022;13:942643.

8. Xie YC, Yao ZH, Yao XL, Pan JZ, Zhang SF, Zhang Y, et al. Glucagon-Like Peptide-2 Receptor is Involved in Spatial Cognitive Dysfunction in Rats After Chronic Cerebral Hypoperfusion. Journal of Alzheimer's disease : JAD. 2018;66(4):1559-76.

9. Cui SY, Yang MX, Zhang YH, Zheng V, Zhang HT, Gurney ME, et al. Protection from Amyloid β Peptide-Induced Memory, Biochemical, and Morphological Deficits by a Phosphodiesterase-4D Allosteric Inhibitor. The Journal of pharmacology and experimental therapeutics. 2019;371(2):250-9.

10. Potashkin JA, Bottero V, Santiago JA, Quinn JP. Bioinformatic Analysis Reveals Phosphodiesterase 4D-Interacting Protein as a Key Frontal Cortex Dementia Switch Gene. Int J Mol Sci. 2020;21(11).

11. Franceschi M, Comola M, Nemni R, Pinto P, Iannaccone S, Smirne S, et al. Neuron-binding antibodies in Alzheimer's disease and Down's syndrome. J Gerontol. 1989;44(4):M128-30.

12. El Khoury JB, Moore KJ, Means TK, Leung J, Terada K, Toft M, et al. CD36 mediates the innate host response to beta-amyloid. The Journal of experimental medicine. 2003;197(12):1657-66.

13. Lu AT, Hannon E, Levine ME, Hao K, Crimmins EM, Lunnon K, et al. Genetic variants near MLST8 and DHX57 affect the epigenetic age of the cerebellum. Nature communications. 2016;7:10561.

14. Heredia L, Helguera P, de Olmos S, Kedikian G, Solá Vigo F, LaFerla F, et al. Phosphorylation of actin-depolymerizing factor/cofilin by LIM-kinase mediates amyloid beta-induced degeneration: a potential mechanism of neuronal dystrophy in Alzheimer's disease. The Journal of neuroscience : the official journal of the Society for Neuroscience. 2006;26(24):6533-42.

15. Yeşil G, Aralaşmak A, Akyüz E, İçağasıoğlu D, Uygur Şahin T, Bayram Y. Expanding the Phenotype of Homozygous KCNMA1 Mutations; Dyskinesia, Epilepsy, Intellectual Disability, Cerebellar and Corticospinal Tract Atrophy. Balkan Med J. 2018;35(4):336-9.

16. Tsai AP, Dong C, Lin PB, Messenger EJ, Casali BT, Moutinho M, et al. PLCG2 is associated with the inflammatory response and is induced by amyloid plaques in Alzheimer's disease. Genome medicine. 2022;14(1):17.

17. van der Lee SJ, Conway OJ, Jansen I, Carrasquillo MM, Kleineidam L, van den Akker E, et al. A nonsynonymous mutation in PLCG2 reduces the risk of Alzheimer's disease, dementia with Lewy bodies and frontotemporal dementia, and increases the likelihood of longevity. Acta neuropathologica. 2019;138(2):237-50.

18. Gracie S, Sengupta N, Ferreira C, Pemberton J, Anderson I, Wang X, et al. De novo loss-of-function variant in PTDSS1 is associated with developmental delay. American journal of medical genetics Part A. 2022;188(6):1739-45.

19. Kochmanski J, Kuhn NC, Bernstein AI. Parkinson's disease-associated, sex-specific changes in DNA methylation at PARK7 (DJ-1), SLC17A6 (VGLUT2), PTPRN2 (IA-2β), and NR4A2 (NURR1) in cortical neurons. NPJ Parkinsons Dis. 2022;8(1):120.

20. Gordon HM, Majithia N, MacDonald PE, Fox JEM, Sharma PR, Byrne FL, et al. STEAP4 expression in human islets is associated with differences in body mass index, sex, HbA1c, and inflammation. Endocrine. 2017;56(3):528-37.

21. Wågsäter D, Björk H, Zhu C, Björkegren J, Valen G, Hamsten A, et al. ADAMTS-4 and -8 are inflammatory regulated enzymes expressed in macrophage-rich areas of human atherosclerotic plaques. Atherosclerosis. 2008;196(2):514-22.

22. Kang H, Han AR, Zhang A, Jeong H, Koh W, Lee JM, et al. GolpHCat (TMEM87A), a unique voltage-dependent cation channel in Golgi apparatus, contributes to Golgi-pH maintenance and hippocampus-dependent memory. Nature communications. 2024;15(1):5830.

23. Mirza Z, Rajeh N. Identification of Electrophysiological Changes in Alzheimer's Disease: A Microarray Based Transcriptomics and Molecular Pathway Analysis Study. CNS & neurological disorders drug targets. 2017;16(9):1027-38.

24. Wang X, Qi Y, Zhou X, Zhang G, Fu C. Alteration of scaffold: Possible role of MACF1 in Alzheimer's disease pathogenesis. Medical hypotheses. 2019;130:109259.

25. Pol-Fuster J, Cañellas F, Ruiz-Guerra L, Medina-Dols A, Bisbal-Carrió B, Asensio V, et al. Familial Psychosis Associated With a Missense Mutation at MACF1 Gene Combined With the Rare Duplications DUP3p26.3 and DUP16q23.3, Affecting the CNTN6 and CDH13 Genes. Front Genet. 2021;12:622886.

26. Anazi S, Maddirevula S, Faqeih E, Alsedairy H, Alzahrani F, Shamseldin HE, et al. Clinical genomics expands the morbid genome of intellectual disability and offers a high diagnostic yield. Mol Psychiatry. 2017;22(4):615-24.

27. Bisht I, Ambasta RK, Kumar P. An integrated approach to unravel a putative crosstalk network in Alzheimer's disease and Parkinson's disease. Neuropeptides. 2020;83:102078.

28. Lobanov SV, McAllister B, McDade-Kumar M, Landwehrmeyer GB, Orth M, Rosser AE, et al. Huntington's disease age at motor onset is modified by the tandem hexamer repeat in TCERG1. NPJ Genom Med. 2022;7(1):53.

29. Sánchez-Lanzas R, Castaño JG. Mitochondrial LonP1 protease is implicated in the degradation of unstable Parkinson's disease-associated DJ-1/PARK 7 missense mutants. Sci Rep. 2021;11(1):7320.

30. Jiang S, Zhang CY, Tang L, Zhao LX, Chen HZ, Qiu Y. Integrated Genomic Analysis Revealed Associated Genes for Alzheimer's Disease in APOE4 Non-Carriers. Current Alzheimer research. 2019;16(8):753-63.

31. Lordén G, Newton AC. Conventional protein kinase C in the brain: repurposing cancer drugs for neurodegenerative treatment? Neuronal Signal. 2021;5(4):Ns20210036.

32. Takahashi H, Adachi N, Shirafuji T, Danno S, Ueyama T, Vendruscolo M, et al. Identification and characterization of PKCγ, a kinase associated with SCA14, as an amyloidogenic protein. Human molecular genetics. 2015;24(2):525-39.

33. Liu M, Sun A, Shin EJ, Liu X, Kim SG, Runyons CR, et al. Expression of microsomal epoxide hydrolase is elevated in Alzheimer's hippocampus and induced by exogenous beta-amyloid and trimethyl-tin. The European journal of neuroscience. 2006;23(8):2027-34.

34. Deming Y, Li Z, Kapoor M, Harari O, Del-Aguila JL, Black K, et al. Genome-wide association study identifies four novel loci associated with Alzheimer's endophenotypes and disease modifiers. Acta neuropathologica. 2017;133(5):839-56.

35. Nadeem MS, Hosawi S, Alshehri S, Ghoneim MM, Imam SS, Murtaza BN, et al. Symptomatic, Genetic, and Mechanistic Overlaps between Autism and Alzheimer's Disease. Biomolecules. 2021;11(11).

36. Zhu M, Jia L, Li F, Jia J. Identification of KIAA0513 and Other Hub Genes Associated With Alzheimer Disease Using Weighted Gene Coexpression Network Analysis. Front Genet. 2020;11:981.

37. Mallah K, Quanico J, Raffo-Romero A, Cardon T, Aboulouard S, Devos D, et al. Mapping Spatiotemporal Microproteomics Landscape in Experimental Model of Traumatic Brain Injury Unveils a link to Parkinson's Disease. Molecular & cellular proteomics : MCP. 2019;18(8):1669-82.

38. Orlandi C, Sutton LP, Muntean BS, Song C, Martemyanov KA. Homeostatic cAMP regulation by the RGS7 complex controls depression-related behaviors. Neuropsychopharmacology : official publication of the American College of Neuropsychopharmacology. 2019;44(3):642-53.

39. Zheng JJ, Li WX, Liu JQ, Guo YC, Wang Q, Li GH, et al. Low expression of aging-related NRXN3 is associated with Alzheimer disease: A systematic review and meta-analysis. Medicine. 2018;97(28):e11343.

40. Li WX, Li GH, Tong X, Yang PP, Huang JF, Xu L, et al. Systematic metabolic analysis of potential target, therapeutic drug, diagnostic method and animal model applicability in three neurodegenerative diseases. Aging. 2020;12(10):9882-914.

41. De Jager PL, Srivastava G, Lunnon K, Burgess J, Schalkwyk LC, Yu L, et al. Alzheimer's disease: early alterations in brain DNA methylation at ANK1, BIN1, RHBDF2 and other loci. Nature neuroscience. 2014;17(9):1156-63.

42. Yao M, Yang Q, Lian M, Su P, Cui X, Ren T, et al. Generation of Dip2a homozygous knockout murine ES cell line IBMSe001-A-1 via CRISPR/Cas9 technology. Stem cell research. 2020;45:101778.

43. Hassan WM, Merin DA, Fonte V, Link CD. AIP-1 ameliorates beta-amyloid peptide toxicity in a Caenorhabditis elegans Alzheimer's disease model. Human molecular genetics. 2009;18(15):2739-47.

44. Wang J, Chen P, Hu B, Cai F, Xu Q, Pan S, et al. Distinct effects of SDC3 and FGFRL1 on selective neurodegeneration in AD and PD. FASEB journal : official publication of the Federation of American Societies for Experimental Biology. 2023;37(2):e22773.

45. Xu H, Jia J. Immune-Related Hub Genes and the Competitive Endogenous RNA Network in Alzheimer's Disease. Journal of Alzheimer's disease : JAD. 2020;77(3):1255-65.

46. Zou C, Su L, Pan M, Chen L, Li H, Zou C, et al. Exploration of novel biomarkers in Alzheimer's disease based on four diagnostic models. Front Aging Neurosci. 2023;15:1079433.

47. Lehallier B, Shokhirev MN, Wyss-Coray T, Johnson AA. Data mining of human plasma proteins generates a multitude of highly predictive aging clocks that reflect different aspects of aging. Aging cell. 2020;19(11):e13256.

48. Bereczki E, Branca RM, Francis PT, Pereira JB, Baek JH, Hortobágyi T, et al. Synaptic markers of cognitive decline in neurodegenerative diseases: a proteomic approach. Brain : a journal of neurology. 2018;141(2):582-95.

49. Wu XL, Huang H, Huang YY, Yuan JX, Zhou X, Chen YM. Reduced Pumilio-2 expression in patients with temporal lobe epilepsy and in the lithium-pilocarpine induced epilepsy rat model. Epilepsy & behavior : E&B. 2015;50:31-9.

50. Lauridsen JB, Johansen JL, Rekling JC, Thirstrup K, Moerk A, Sager TN. Regulation of the Bcas1 and Baiap3 transcripts in the subthalamic nucleus in mice recovering from MPTP toxicity. Neuroscience research. 2011;70(3):269-76.

51. Walton E, Pingault JB, Cecil CA, Gaunt TR, Relton CL, Mill J, et al. Epigenetic profiling of ADHD symptoms trajectories: a prospective, methylome-wide study. Mol Psychiatry. 2017;22(2):250-6.

52. Wang X, Lopez OL, Sweet RA, Becker JT, DeKosky ST, Barmada MM, et al. Genetic determinants of disease progression in Alzheimer's disease. Journal of Alzheimer's disease : JAD. 2015;43(2):649-55.

53. Su W, Zhou Q, Wang Y, Chishti A, Li QQ, Dayal S, et al. Deletion of the Capn1 Gene Results in Alterations in Signaling Pathways Related to Alzheimer's Disease, Protein Quality Control and Synaptic Plasticity in Mouse Brain. Front Genet. 2020;11:334.

54. Gan-Or Z, Bouslam N, Birouk N, Lissouba A, Chambers DB, Vérièpe J, et al. Mutations in CAPN1 Cause Autosomal-Recessive Hereditary Spastic Paraplegia. American journal of human genetics. 2016;98(5):1038-46.

55. Schmeisser MJ, Kühl SJ, Schoen M, Beth NH, Weis TM, Grabrucker AM, et al. The Nedd4-binding protein 3 (N4BP3) is crucial for axonal and dendritic branching in developing neurons. Neural Dev. 2013;8:18.

56. Mbefo MK, Paleologou KE, Boucharaba A, Oueslati A, Schell H, Fournier M, et al. Phosphorylation of synucleins by members of the Polo-like kinase family. The Journal of biological chemistry. 2010;285(4):2807-22.

57. Pérez-Roca L, Prada-Dacasa P, Segú-Vergés C, Gámez-Valero A, Serrano-Muñoz MA, Santos C, et al. Glucocerebrosidase regulators SCARB2 and TFEB are up-regulated in Lewy body disease brain. Neuroscience letters. 2019;706:164-8.

58. Leandro GS, Evangelista AF, Lobo RR, Xavier DJ, Moriguti JC, Sakamoto-Hojo ET. Changes in Expression Profiles Revealed by Transcriptomic Analysis in Peripheral Blood Mononuclear Cells of Alzheimer's Disease Patients. Journal of Alzheimer's disease : JAD. 2018;66(4):1483-95.

59. Kim H, Lim J, Bao H, Jiao B, Canon SM, Epstein MP, et al. Rare variants in MYH15 modify amyotrophic lateral sclerosis risk. Human molecular genetics. 2019;28(14):2309-18.

60. Gao H, Tao Y, He Q, Song F, Saffen D. Functional enrichment analysis of three Alzheimer's disease genome-wide association studies identities DAB1 as a novel candidate liability/protective gene. Biochemical and biophysical research communications. 2015;463(4):490-5.

61. Kim D, Basile AO, Bang L, Horgusluoglu E, Lee S, Ritchie MD, et al. Knowledge-driven binning approach for rare variant association analysis: application to neuroimaging biomarkers in Alzheimer's disease. BMC medical informatics and decision making. 2017;17(Suppl 1):61.

62. Moore K, McKnight AJ, Craig D, O'Neill F. Epigenome-wide association study for Parkinson's disease. Neuromolecular Med. 2014;16(4):845-55.

63. Lancour D, Dupuis J, Mayeux R, Haines JL, Pericak-Vance MA, Schellenberg GC, et al. Analysis of brain region-specific co-expression networks reveals clustering of established and novel genes associated with Alzheimer disease. Alzheimer's research & therapy. 2020;12(1):103.

64. Jin F, Xi Y, Xie D, Wang Q. Comprehensive analysis reveals a 5-gene signature and immune cell infiltration in Alzheimer's disease with qPCR validation. Front Genet. 2022;13:913535.

65. Gao F, Zhang J, Ni T, Lin N, Lin H, Luo H, et al. Herpud1 deficiency could reduce amyloid-β40 expression and thereby suppress homocysteine-induced atherosclerosis by blocking the JNK/AP1 pathway. J Physiol Biochem. 2020;76(3):383-91.

66. Riise J, Plath N, Pakkenberg B, Parachikova A. Aberrant Wnt signaling pathway in medial temporal lobe structures of Alzheimer's disease. Journal of neural transmission (Vienna, Austria : 1996). 2015;122(9):1303-18.

67. Bertini V, Valetto A, Azzarà A, Legitimo A, Saggese G, Consolini R, et al. A Case of 22q11 Deletion Syndrome (22q11DS) with a Panayiotopoulos Epileptic Pattern: Are Additional Copy-Number Variations a Possible Second Hit in Modulating the 22q11DS Phenotype? Front Pediatr. 2017;5:48.

68. Kim J, Zhang Y, Pan W. Powerful and Adaptive Testing for Multi-trait and Multi-SNP Associations with GWAS and Sequencing Data. Genetics. 2016;203(2):715-31.

69. Ye X, Chen L, Wang H, Peng S, Liu M, Yao L, et al. Genetic inhibition of PDK1 robustly reduces plaque deposition and ameliorates gliosis in the 5×FAD mouse model of Alzheimer's disease. Neuropathol Appl Neurobiol. 2022;48(7):e12839.

70. Díaz-Casado E, Gómez-Nieto R, de Pereda JM, Muñoz LJ, Jara-Acevedo M, López DE. Analysis of gene variants in the GASH/Sal model of epilepsy. PLoS One. 2020;15(3):e0229953.

71. Chen S, Chang Y, Li L, Acosta D, Li Y, Guo Q, et al. Spatially resolved transcriptomics reveals genes associated with the vulnerability of middle temporal gyrus in Alzheimer's disease. Acta Neuropathol Commun. 2022;10(1):188.

72. Iwama K, Iwata A, Shiina M, Mitsuhashi S, Miyatake S, Takata A, et al. A novel mutation in SLC1A3 causes episodic ataxia. Journal of human genetics. 2018;63(2):207-11.

73. Wang X, Liu L, Jiang X, Saredy J, Xi H, Cueto R, et al. Identification of methylation-regulated genes modulating microglial phagocytosis in hyperhomocysteinemia-exacerbated Alzheimer's disease. Alzheimer's research & therapy. 2023;15(1):164.

74. Daschil N, Obermair GJ, Flucher BE, Stefanova N, Hutter-Paier B, Windisch M, et al. CaV1.2 calcium channel expression in reactive astrocytes is associated with the formation of amyloid-β plaques in an Alzheimer's disease mouse model. Journal of Alzheimer's disease : JAD. 2013;37(2):439-51.

75. Schmied MC, Zehetmayer S, Reindl M, Ehling R, Bajer-Kornek B, Leutmezer F, et al. Replication study of multiple sclerosis (MS) susceptibility alleles and correlation of DNA-variants with disease features in a cohort of Austrian MS patients. Neurogenetics. 2012;13(2):181-7.

76. Orozco D, Edbauer D. FUS-mediated alternative splicing in the nervous system: consequences for ALS and FTLD. Journal of molecular medicine (Berlin, Germany). 2013;91(12):1343-54.

77. Toyn JH, Lin XA, Thompson MW, Guss V, Meredith JE, Jr., Sankaranarayanan S, et al. Viable mouse gene ablations that robustly alter brain Aβ levels are rare. BMC neuroscience. 2010;11:143.

78. Bleasel JM, Hsiao JH, Halliday GM, Kim WS. Increased expression of ABCA8 in multiple system atrophy brain is associated with changes in pathogenic proteins. Journal of Parkinson's disease. 2013;3(3):331-9.

79. Smolen GA, Schott BJ, Stewart RA, Diederichs S, Muir B, Provencher HL, et al. A Rap GTPase interactor, RADIL, mediates migration of neural crest precursors. Genes Dev. 2007;21(17):2131-6.

80. Wang T, Li P, Meng X, Zhang J, Liu Q, Jia C, et al. An integrated pathological research for precise diagnosis of schizophrenia combining LC-MS/(1)H NMR metabolomics and transcriptomics. Clin Chim Acta. 2022;524:84-95.

81. Li Y, Fan H, Sun J, Ni M, Zhang L, Chen C, et al. Circular RNA expression profile of Alzheimer's disease and its clinical significance as biomarkers for the disease risk and progression. The international journal of biochemistry & cell biology. 2020;123:105747.

82. Mohandas N, Loke YJ, Hopkins S, Mackenzie L, Bennett C, Berkovic SF, et al. Evidence for type-specific DNA methylation patterns in epilepsy: a discordant monozygotic twin approach. Epigenomics. 2019;11(8):951-68.

83. Horjus J, van Mourik-Banda T, Heerings MAP, Hakobjan M, De Witte W, Heersema DJ, et al. Whole Exome Sequencing in Multi-Incident Families Identifies Novel Candidate Genes for Multiple Sclerosis. Int J Mol Sci. 2022;23(19).

84. Wagner M, Lévy J, Jung-Klawitter S, Bakhtiari S, Monteiro F, Maroofian R, et al. Loss of TNR causes a nonprogressive neurodevelopmental disorder with spasticity and transient opisthotonus. Genet Med. 2020;22(6):1061-8.

85. Gallardo-Orihuela A, Hervás-Corpión I, Hierro-Bujalance C, Sanchez-Sotano D, Jiménez-Gómez G, Mora-López F, et al. Transcriptional correlates of the pathological phenotype in a Huntington's disease mouse model. Sci Rep. 2019;9(1):18696.

86. Paine I, Posey JE, Grochowski CM, Jhangiani SN, Rosenheck S, Kleyner R, et al. Paralog Studies Augment Gene Discovery: DDX and DHX Genes. American journal of human genetics. 2019;105(2):302-16.

87. Ali Z, Klar J, Jameel M, Khan K, Fatima A, Raininko R, et al. Novel SACS mutations associated with intellectual disability, epilepsy and widespread supratentorial abnormalities. J Neurol Sci. 2016;371:105-11.

88. Liu N, Xu J, Liu H, Zhang S, Li M, Zhou Y, et al. Hippocampal transcriptome-wide association study and neurobiological pathway analysis for Alzheimer's disease. PLoS Genet. 2021;17(2):e1009363.

89. Lin PI, Martin ER, Browning-Large CA, Schmechel DE, Welsh-Bohmer KA, Doraiswamy PM, et al. Parsing the genetic heterogeneity of chromosome 12q susceptibility genes for Alzheimer disease by family-based association analysis. Neurogenetics. 2006;7(3):157-65.

90. Kwiatkowski D, Czarny P, Toma M, Korycinska A, Sowinska K, Galecki P, et al. Association between Single-Nucleotide Polymorphisms of the hOGG1,NEIL1,APEX1, FEN1,LIG1, and LIG3 Genes and Alzheimer's Disease Risk. Neuropsychobiology. 2016;73(2):98-107.

91. Sharma NK, Lebedeva M, Thomas T, Kovalenko OA, Stumpf JD, Shadel GS, et al. Intrinsic mitochondrial DNA repair defects in Ataxia Telangiectasia. DNA repair. 2014;13:22-31.

92. Mews P, Donahue G, Drake AM, Luczak V, Abel T, Berger SL. Acetyl-CoA synthetase regulates histone acetylation and hippocampal memory. Nature. 2017;546(7658):381-6.

93. Hnoonual A, Thammachote W, Tim-Aroon T, Rojnueangnit K, Hansakunachai T, Sombuntham T, et al. Chromosomal microarray analysis in a cohort of underrepresented population identifies SERINC2 as a novel candidate gene for autism spectrum disorder. Sci Rep. 2017;7(1):12096.

94. Wu S, Yang F, Chao S, Wang B, Wang W, Li H, et al. Altered DNA methylome profiles of blood leukocytes in Chinese patients with mild cognitive impairment and Alzheimer's disease. Front Genet. 2023;14:1175864.

95. Janssens J, Philtjens S, Kleinberger G, Van Mossevelde S, van der Zee J, Cacace R, et al. Investigating the role of filamin C in Belgian patients with frontotemporal dementia linked to GRN deficiency in FTLD-TDP brains. Acta Neuropathol Commun. 2015;3:68.

96. Hashimoto R, Nakazawa T, Tsurusaki Y, Yasuda Y, Nagayasu K, Matsumura K, et al. Whole-exome sequencing and neurite outgrowth analysis in autism spectrum disorder. Journal of human genetics. 2016;61(3):199-206.

97. Zhao L, He Z, Zhang D, Wang GT, Renton AE, Vardarajan BN, et al. A Rare Variant Nonparametric Linkage Method for Nuclear and Extended Pedigrees with Application to Late-Onset Alzheimer Disease via WGS Data. American journal of human genetics. 2019;105(4):822-35.

98. Marengo L, Armbrust F, Schoenherr C, Storck SE, Schmitt U, Zampar S, et al. Meprin β knockout reduces brain Aβ levels and rescues learning and memory impairments in the APP/lon mouse model for Alzheimer's disease. Cell Mol Life Sci. 2022;79(3):168.

99. Zhang LH, Wang X, Stoltenberg M, Danscher G, Huang L, Wang ZY. Abundant expression of zinc transporters in the amyloid plaques of Alzheimer's disease brain. Brain Res Bull. 2008;77(1):55-60.

100. Byström S, Ayoglu B, Häggmark A, Mitsios N, Hong MG, Drobin K, et al. Affinity proteomic profiling of plasma, cerebrospinal fluid, and brain tissue within multiple sclerosis. J Proteome Res. 2014;13(11):4607-19.

101. Gns HS, Rajalekshmi SG, Burri RR. Revelation of Pivotal Genes Pertinent to Alzheimer's Pathogenesis: A Methodical Evaluation of 32 GEO Datasets. J Mol Neurosci. 2022;72(2):303-22.

102. Suo Z, Cox AA, Bartelli N, Rasul I, Festoff BW, Premont RT, et al. GRK5 deficiency leads to early Alzheimer-like pathology and working memory impairment. Neurobiology of aging. 2007;28(12):1873-88.

103. Murray PS, Kirkwood CM, Gray MC, Ikonomovic MD, Paljug WR, Abrahamson EE, et al. β-Amyloid 42/40 ratio and kalirin expression in Alzheimer disease with psychosis. Neurobiology of aging. 2012;33(12):2807-16.

104. Granno S, Nixon-Abell J, Berwick DC, Tosh J, Heaton G, Almudimeegh S, et al. Downregulated Wnt/β-catenin signalling in the Down syndrome hippocampus. Sci Rep. 2019;9(1):7322.

105. Yang C, Li S, Ma JX, Li Y, Zhang A, Sun N, et al. Whole Exome Sequencing Identifies a Novel Predisposing Gene, MAPKAP1, for Familial Mixed Mood Disorder. Front Genet. 2019;10:74.

106. Lamont EW, Legault-Coutu D, Cermakian N, Boivin DB. The role of circadian clock genes in mental disorders. Dialogues in clinical neuroscience. 2007;9(3):333-42.

107. Jansen IE, Ye H, Heetveld S, Lechler MC, Michels H, Seinstra RI, et al. Discovery and functional prioritization of Parkinson's disease candidate genes from large-scale whole exome sequencing. Genome Biol. 2017;18(1):22.

108. Scheubert L, Luštrek M, Schmidt R, Repsilber D, Fuellen G. Tissue-based Alzheimer gene expression markers-comparison of multiple machine learning approaches and investigation of redundancy in small biomarker sets. BMC bioinformatics. 2012;13:266.

109. Fan M, Liu Y, Shang Y, Xue Y, Liang J, Huang Z. JADE2 Is Essential for Hippocampal Synaptic Plasticity and Cognitive Functions in Mice. Biological psychiatry. 2022;92(10):800-14.

110. Kramer NJ, Carlomagno Y, Zhang YJ, Almeida S, Cook CN, Gendron TF, et al. Spt4 selectively regulates the expression of C9orf72 sense and antisense mutant transcripts. Science (New York, NY). 2016;353(6300):708-12.

111. Cochet-Bissuel M, Lory P, Monteil A. The sodium leak channel, NALCN, in health and disease. Frontiers in cellular neuroscience. 2014;8:132.

112. Guo D, Peng Y, Wang L, Sun X, Wang X, Liang C, et al. Autism-like social deficit generated by Dock4 deficiency is rescued by restoration of Rac1 activity and NMDA receptor function. Mol Psychiatry. 2021;26(5):1505-19.

113. Farrell K, Kim S, Han N, Iida MA, Gonzalez EM, Otero-Garcia M, et al. Genome-wide association study and functional validation implicates JADE1 in tauopathy. Acta neuropathologica. 2022;143(1):33-53.

114. Wang C, Saar V, Leung KL, Chen L, Wong G. Human amyloid β peptide and tau co-expression impairs behavior and causes specific gene expression changes in Caenorhabditis elegans. Neurobiology of disease. 2018;109(Pt A):88-101.

115. Le Duc D, Giulivi C, Hiatt SM, Napoli E, Panoutsopoulos A, Harlan De Crescenzo A, et al. Pathogenic WDFY3 variants cause neurodevelopmental disorders and opposing effects on brain size. Brain : a journal of neurology. 2019;142(9):2617-30.

116. Triaca V, Sposato V, Bolasco G, Ciotti MT, Pelicci P, Bruni AC, et al. NGF controls APP cleavage by downregulating APP phosphorylation at Thr668: relevance for Alzheimer's disease. Aging cell. 2016;15(4):661-72.

117. Ni H, Xu M, Zhan GL, Fan Y, Zhou H, Jiang HY, et al. The GWAS Risk Genes for Depression May Be Actively Involved in Alzheimer's Disease. Journal of Alzheimer's disease : JAD. 2018;64(4):1149-61.

118. Griñán-Ferré C, Izquierdo V, Otero E, Puigoriol-Illamola D, Corpas R, Sanfeliu C, et al. Environmental Enrichment Improves Cognitive Deficits, AD Hallmarks and Epigenetic Alterations Presented in 5xFAD Mouse Model. Frontiers in cellular neuroscience. 2018;12:224.

119. Yin KJ, Fan Y, Hamblin M, Zhang J, Zhu T, Li S, et al. KLF11 mediates PPARγ cerebrovascular protection in ischaemic stroke. Brain : a journal of neurology. 2013;136(Pt 4):1274-87.

120. Ray M, Ruan J, Zhang W. Variations in the transcriptome of Alzheimer's disease reveal molecular networks involved in cardiovascular diseases. Genome Biol. 2008;9(10):R148.

121. Niu L, Zhang F, Xu X, Yang Y, Li S, Liu H, et al. Chronic sleep deprivation altered the expression of circadian clock genes and aggravated Alzheimer's disease neuropathology. Brain Pathol. 2022;32(3):e13028.

122. Yang Z, Matsumoto A, Nakayama K, Jimbo EF, Kojima K, Nagata K, et al. Circadian-relevant genes are highly polymorphic in autism spectrum disorder patients. Brain & development. 2016;38(1):91-9.

123. Shkodina AD, Tan SC, Hasan MM, Abdelgawad M, Chopra H, Bilal M, et al. Roles of clock genes in the pathogenesis of Parkinson's disease. Ageing research reviews. 2022;74:101554.

124. Pathak GA, Silzer TK, Sun J, Zhou Z, Daniel AA, Johnson L, et al. Genome-Wide Methylation of Mild Cognitive Impairment in Mexican Americans Highlights Genes Involved in Synaptic Transport, Alzheimer's Disease-Precursor Phenotypes, and Metabolic Morbidities. Journal of Alzheimer's disease : JAD. 2019;72(3):733-49.

125. Lei D, Jin X, Wen L, Dai H, Ye Z, Wang G. bmp3 is Required for Integrity of Blood Brain Barrier by Promoting Pericyte Coverage in Zebrafish Embryos. Curr Mol Med. 2017;17(4):298-303.
